# Considerations for improved performance of competition association assays analysed with the Motulsky–Mahan's “kinetics of competitive binding” model

**DOI:** 10.1111/bph.14841

**Published:** 2019-12-26

**Authors:** Victoria Georgi, Alexey Dubrovskiy, Stephan Steigele, Amaury E. Fernández‐Montalván

**Affiliations:** ^1^ Drug Discovery, Pharmaceuticals Bayer AG Berlin Germany; ^2^ Research and Development Genedata AG Basel Switzerland; ^3^ Software Engineering Google Inc. Zürich Switzerland; ^4^ Compound Screening Institut de Recherches Servier Croissy‐sur‐Seine France

## Abstract

**Background and Purpose:**

Target engagement dynamics can influence drugs' pharmacological effects. Kinetic parameters for drug:target interactions are often quantified by evaluating competition association experiments—measuring simultaneous protein binding of labelled tracers and unlabelled test compounds over time—with Motulsky–Mahan's “kinetics of competitive binding” model. Despite recent technical improvements, the current assay formats impose practical limitations to this approach. This study aims at the characterisation, understanding and prevention of these experimental constraints, and associated analytical challenges.

**Experimental Approach:**

Monte Carlo simulations were used to run virtual kinetic and equilibrium tracer binding and competition experiments in both normal and perturbed assay conditions. Data were fitted to standard equations derived from the mass action law (including Motulsky–Mahan's) and to extended versions aiming to cope with frequently observed deviations of the canonical traces. Results were compared to assess the precision and accuracy of these models and identify experimental factors influencing their performance.

**Key Results:**

Key factors influencing the precision and accuracy of the Motulsky–Mahan model are the interplay between compound dissociation rates, measurement time and interval frequency, tracer concentration and binding kinetics and the relative abundance of equilibrium complexes in vehicle controls. Experimental results produced recommendations for better design of tracer characterisation experiments and new strategies to deal with systematic signal decay.

**Conclusions and Implications:**

Our data advances our comprehension of the Motulsky–Mahan kinetics of competitive binding models and provides experimental design recommendations, data analysis tools, and general guidelines for its practical application to in vitro pharmacology and drug screening.

**Abbreviations**
BK: binding kinetics
CV: coefficient of variation
k_off_: dissociation rate
k_on_: association rate
kPCA: kinetic probe competition assay
MC (analyses/simulation): Monte Carlo (analyses/simulation)
RT: residence time

**What is already known**

- The Motulsky–Mahan “kinetics of competitive binding” model allows rate constant determination for drug:target interactions.
- Current configurations of kinetic competition association assays can present challenges for the Motulsky–Mahan model.

**What this study adds**

- In silico reproduction and characterisation of experimental challenges observed under typical competition association assay conditions.
- Recommendations to increase Motulsky–Mahan model performance fitting kinetic traces while keeping reasonable assay throughput.

**What is the clinical significance**

- Target binding kinetics modulate safety and efficacy profiles of drugs, thereby differentiating their clinical outcomes.
- This study enables more precise and accurate kinetic parameter quantification for existing and novel medicines.

## 1 INTRODUCTION

The kinetics of drug interactions with their targets are thought to influence pharmacological effects in patients, especially in situations where binding equilibrium is not reached (Copeland, 2016; Copeland, Pompliano, & Meek, 2006; Paton, 1960; Paton, 1961; Swinney, 2006). Long target residence times can lead to occupancies outlasting the pharmacokinetics of a compound and, consequently, to sustained efficacy after the free drug has been cleared from the system. Moreover, differentiated kinetic profiles for primary and off‐targets can result in kinetic selectivity despite similar steady‐state affinities for both targets (Copeland et al., 2006; Dahl & Akerud, 2013). Thus, characterisation of screening hits, leads, and candidate compounds regarding their target binding kinetics (BK) has become a standard in drug discovery laboratories.

The growing interest in BK has promoted the development of assay technologies dedicated to measure association (k_on_) and dissociation (k_off_) rate constants, as well as binding affinities, (*K*_D_ = k_off_/k_on_; Antoine et al., 2016; de Witte et al., 2018; Guo, Mulder‐Krieger, IJzerman, & Heitman, 2012; Schiele, Ayaz, & Fernández‐Montalván, 2015; Stoddard et al., 2018; Stoddart et al., 2015; Swinney et al., 2014; Sykes et al., 2017; Xia, de Vries, IJzerman, & Heitman, 2016). Among them, competition association assays, monitoring the simultaneous binding of a labelled tracer (we have referred to the labelled compound as tracer, although any type of fluorescent or luminescent probes and radioactive tracers can be used for competition association assays) and an unlabelled compound to the target molecule, have gained popularity due to their relatively high throughput and ability to deal with both soluble and membrane‐bound proteins (Schiele et al., 2015; Stoddart et al., 2015; Xia et al., 2016). In these assays, the rate constants for test compounds' interactions with their molecular targets are typically obtained from fitting the experimental data to the “kinetics of competitive binding” equation initially reported by Motulsky & Mahan (1984). This analytical procedure uses a differential equation system where the compound dose (I), tracer concentration (L), tracer k_on_ (k_1_), and tracer k_off_ (k_2_) values are known (fix) parameters, to solve for the unknown test compounds' k_on_ (k_3_) and k_off_ (k_4_) values. To this end, k_1_ and k_2_ are determined in direct binding experiments ideally performed under similar conditions as the competition assays.

In recent years, our laboratory routinely performed high throughput homogenous TR‐FRET‐based competition association assays (also known as kPCA: kinetic probe competition assay, Schiele et al., 2015) to evaluate hundreds of compounds interacting with several dozens of targets (see Bosma et al., 2019; de Witte et al., 2018; Georgi et al., 2018; Heroven et al., 2018; Nederpelt et al., 2016; Schiele et al., 2015). Typically, k_on_, k_off_, and derived affinities (*K*_D,kin_) of test compounds showed excellent agreement among replicates, and with reference values. However, under our experimental conditions, we encountered two recurring situations in which analysing data with the Motulsky–Mahan model became challenging: The first issue (*Case 1*) was characterised by k_on_ values being determined precisely in replicate measurements, but with a high variability of the corresponding k_off_ and *K*_D,kin_ (CV > 50%). In these examples, *K*_D,kin_ always differ from the steady‐state affinity values (*K*_D,eq_) calculated from an independent equilibrium probe competition assay (ePCA). The second problem (*Case 2*) manifested itself by k_on_ and k_off_ parameters determined with poor precision (CV > 50%) despite acceptable data quality, and the fact that the corresponding *K*_D,kin_ was consistent with *K*_D,eq_. While in *Case 1*, the k_off_ was obtained by multiplying the on‐rate by the *K*_Deq_ from ePCAs (Schiele et al., 2015; Georgi et al., 2018) no meaningful BK values could be extracted from measurements described by *Case 2* (Georgi et al., 2018).

These are examples of decreased precision and accuracy and here it is necessary to define what we mean by these terms: Precision is the closeness of a set of measured replicate values to each other, and accuracy is the agreement of the measured value compared to the true value (JCGM200:2012 International vocabulary of metrology – Basic and general concepts and associated terms (VIM).3 edn.). The true value is the value that would be obtained by a perfect measurement but is indeterminable in reality. Nonetheless, the mean values acquired under repeatability conditions (=same assay components, measuring, and evaluation procedure) will be similar to the true value as long as there is no systematic error.

We were motivated by such examples of decreased precision and accuracy, when fitting assay data to the Motulsky–Mahan “kinetics of competitive binding” model, to ask whether they were random or reproducible events, linked to specific experimental conditions. To address this question, we generated and evaluated thousands of simulated data points in a variety of assay set‐ups via Monte Carlo (MC) simulations. The results described here provide explanations for the limitations described above, as well as recommendations for better design and analysis of these experiments, especially if they are run using the standard instrumentation of current drug screening laboratories.

## 2 METHODS

### 2.1 MC analyses

To test the performance of the non‐linear multiparametric Motulsky–Mahan model within the parameter space, we performed MC simulations (Metropolis & Ulam, 1949) of thousands of pseudo‐interactions with known input parameters, including the association and dissociation rates of the compounds, and with a random scatter in the measured signals using GraphPad Prism versions 6.07 and 7.00 for Windows (GraphPad Software, San Diego California USA, http://www.graphpad, RRID:SCR_002798). Our kPCA set‐up (described in Schiele et al., 2015) served as reference for the experimental parameters, and—whenever applicable—deviations from the standard procedure were introduced as indicated in Table S1 to simulate systematic or random errors. These in silico experiments were subsequently evaluated by global curve fitting of the obtained signal traces to Motulsky–Mahan's “kinetics of competitive binding” model (Motulsky & Mahan, 1984) or others, depending on the scope of the simulation. For assessment of accuracy, we calculated the relative error of the output variables compared to the true value (input variable). For evaluation of precision, we calculated the coefficient of variation (CV) for all data obtained under repeatability conditions. Details of each pseudo‐experiment are specified in Table S1, as well as in the corresponding figures. In addition, example GraphPad Prism files with the simulations are provided as Data S4 and Data S5 in the Supporting Information file.

### 2.2 Models and equations

Most mathematical models used for our analyses are readily available in GraphPad Prism, and some were generated using the software's equation editing tool. Induced fit and irreversible interactions were modelled with COPASI 4.1.9 (Hoops et al., 2006), RRID:SCR_014260 and are provided as Data S2 and Data S3 in the Supporting Information file. Previously described models and their literature sources are described in the supplementary methods section. Models generated for this study are described below.

Normalised Motulsky–Mahan “kinetics of competitive binding” model (this study):

$$\begin{array}{l} \hat{y}\left(t>0\right)=\left(1-\frac{K_{A}}{\text{Diff}\left(1-e^{-K_{A}t}\right)}\;\left(\frac{k_{4}\text{Diff}}{K_{F}K_{S}}+\frac{k_{4}-K_{F}}{K_{F}}\;e^{-K_{F}t}-\frac{k_{4}-K_{S}}{K_{S}}\;e^{-K_{S}t}\right)\right)\times 100\%\\ \hat{y}\left(t=0\right)=0 \end{array}$$

with

$\begin{array}{l} K_{A}=k_{1}\times L+k_{2}\\ K_{B}=k_{3}\times I+k_{4}\\ \mathrm{KF}=0.5\left(\mathrm{KA}+\mathrm{KB}+\sqrt{{\left(K_{A}-K_{B}\right)}^{2}+4\times k1\times k3\times L\times I}\right)\\ \mathrm{KS}=\;0.5\;\left(\mathrm{KA}\;+\mathrm{KB}–\sqrt{{\left(K_{A}-K_{B}\right)}^{2}+4\times k1\times k3\times L\times I}\right)\\ \text{Diff}=K_{F}–K_{S}, \end{array}$where ŷ: normalised binding signal [%]; t: time [s]; k_1_, k_2_, L: association rate [M^−1^ s^−1^], dissociation rate [s^−1^], and concentration [M] of the tracer, k_3_, I, k_4_: association rate [M^−1^ s^−1^], concentration [M], and dissociation rate [s^−1^] of the unlabelled compound.

With parameters constrains: k_1_, L, k_2_, I constrained to constant values as used in experiment or determined during assay development respectively. Shared parameters (between concentration traces) for global fit: k_3_, k_4_.

Relative (percentage) amount of target: tracer equilibrium reached (this study):

The percentage of equilibrium reached was approximated as follows:

$$\%\mathrm{eq}\;\text{reached}=\left(1-e^{-t\times \left(k_{\mathrm{on}}\times L+k_{\mathrm{off}}\right)}\right)\times 100\%,$$

where %eq: percentage of equilibrium reached at time t = binding signal at time t divided by binding signal at equilibrium multiplied by 100%; t: observation time [s]; k_on_, L, k_off_: association rate [M^−1^ s^−1^], concentration [M], and dissociation rate [s^−1^] of compound.

### 2.3 Data normalisation and statistics

For the normalised “Motulsky–Mahan” model, kinetic traces were normalised as percentage of tracer binding (0%: no tracer binding = background signal = maximum inhibition control; 100%: tracer binding control = no compound binding) and inverted to obtain percentage of compound binding.

Precision was quantified by determination of the CV, which is the quotient of the SD and the mean: CV [%] = SD/mean × 100%.

Accuracy was quantified as relative error of the determined mean value compared to the input value for the simulations:

$$\text{relative error}\;\left[\%\right]=\frac{‘\text{input value}’–\text{mean}\left(‘\text{calculated values}’\right)}{‘\text{input value}’}\times 100\%.$$

Z'‐factor was calculated as described below (Zhang, Chung, & Oldenburg, 1999). The mean of all Z'‐factors at each time point at equilibrium of the BK assay was used to approximate the Z'‐factor of the assay.

$$Z'-\text{factor}=1–3\left({\mathrm{SD}}_{p}+{\mathrm{SD}}_{n}\right)/|{\text{mean}}_{p}-{\text{mean}}_{n}|,$$

where SD_p_, SD_n_, mean_p_, mean_n_: SD and mean of positive p (here: 100% tracer binding) and negative n (background signal, here: 0% tracer binding) controls.

The binding parameters obtained by the two ‘signal decay’ methods described in the supplementary method section (multiplication with signal drift term or normalisation of the “kinetics of competitive binding” equation) were compared to literature radioligand binding data by (a) Spearman correlation calculations (coefficient *r* and two‐tailed *P* value) and (b) Bland–Altman analysis (Bland & Altman, 1986, 1990; mean log differences) using GraphPad Prism.

### 2.4 Nomenclature of targets and ligands

Key protein targets and ligands in this article are hyperlinked to corresponding entries in http://www.guidetopharmacology.org, the common portal for data from the IUPHAR/BPS Guide to PHARMACOLOGY (Harding et al., 2018), and are permanently archived in the Concise Guide to PHARMACOLOGY 2017/18 (Alexander et al., 2017).

## 3 RESULTS

### 3.1 Precision and accuracy of rate constant determination is significantly influenced by target residence times of test compounds'

The *Case 1* described in the introduction prompted us to ask if on‐rates are precisely fitted when the corresponding off‐rates and affinities cannot be determined; the underlying question being whether it is justified to calculate off‐rates as proposed in previous work (Georgi et al., 2018; Schiele et al., 2015). Likewise, to better understand if and to what extent *Case 2* results can be trusted, we decided to explore the range of rate constants that can be quantified with acceptable precision and accuracy under our experimental conditions for competition association assays.

**Figure 1:**
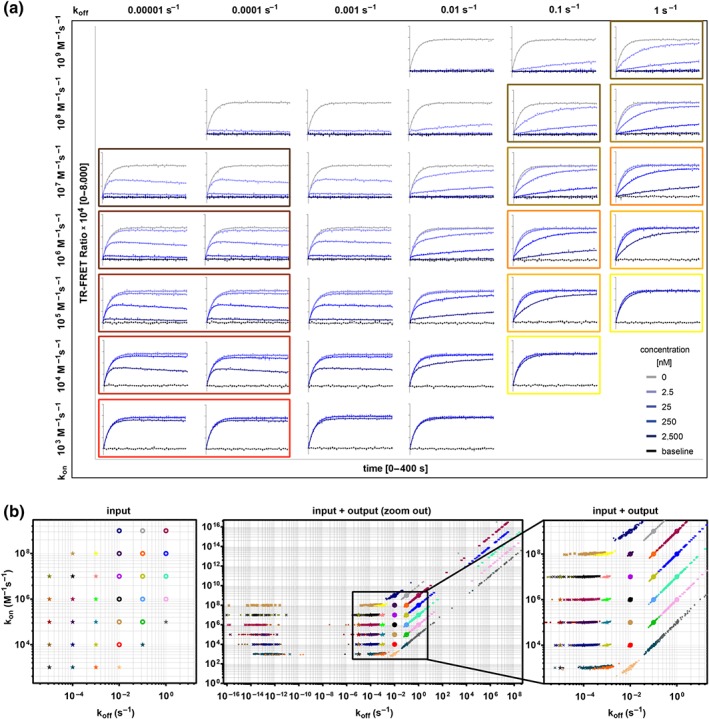
In silico evaluation of the Motulsky–Mahan model performance fitting a range of rate constants measured with the same experimental conditions. (a) Representative graphs of simulated competitive tracer‐compound association traces for 35 virtual compounds with different on‐ and off‐rates. The simulation considered an observation time of 400 s, a measuring interval of 10 s, and 12.5‐nM tracer with a k_on_ = 2.56 × 10^6^ M^−1^ s^−1^ and a k_off_ = 1.67 × 10^−3^ s^−1^. The corresponding fit curves derived from the “kinetics of competitive binding” equation are indicated as solid lines. (b) Monte Carlo simulations and analyses shown in Panel (a) were performed 100 times for each compound. The rate plots represent the input binding kinetic parameters (left panel, and large symbols in all plots) for the 35 compounds (one colour per compound) as used for the simulation along with the corresponding output rates (small symbols in middle and right graphs) calculated by using the Motulsky–Mahan model. The diagonals in the plots correspond to the isoaffinity lines. The right panel zooms into the range of the input parameters, which span a physiologically meaningful range of the binding parameters. In contrast, the middle panel allows visualisation of all generated output parameters

The results of the corresponding MC analyses (described in Table S1, Experiment 1) are shown in Figure 1: Panel (a) displays examples of the MC generated signal traces and their fits to the “kinetics of competitive binding” equation, and Figure 1b shows a rate plot containing both the input values for the 35 compounds and the corresponding output parameters after MC simulations. Inspection of these graphs revealed that for off‐rates between 0.1 and 0.001 s^−1^, the binding parameters determined are in agreement with the expected—that is, input—values (mean relative errors <1.6% and mean CV < 18% for on‐rates, off‐rates, and affinities). Exceptions were observed if the compound‐tracer binding traces did not contain sufficient kinetic information: that is, if all traces were similar to the low‐ (background) or high‐ (tracer binding in vehicle) signal control traces. Prominent outliers were interactions with k_on_ [M^−1^ s^−1^] − k_off_ [s^−1^] combinations of 10^4^–0.1 and 10^9^–0.01 respectively. Further simulations showed that this particular problem is considerably alleviated by using at least 10‐fold lower or higher doses of test compounds (not shown).

Figure 1a also shows that there is an excellent overlap of measured and fitted competitive association traces for compounds sharing the same on‐rate and with dissociation rates of 0.0001 s^−1^ or slower. Consequently, the on‐rates obtained are consistent with the input data, with relative errors of <4% and CV < 7%, but the affinities and off‐rates are calculated with a significant lack of precision and accuracy (errors and CVs of >>500% and >75% respectively, Figure 1b). The fit results for dissociation rates and affinities often drift to extremely low values (Figure 1b), showing exactly the same behaviour as the real life examples represented by *Case 1*. These results suggest that on‐rates in this situation are determined with high precision and accuracy and that the reason why off‐rates and affinities cannot be determined is most likely related to the long residence times of the test compounds. In the following sections, we will use the term “slow off‐rate problem” to describe this in silico reconstitution of real life's *Case 1*.

Compounds placed along an isoaffinity diagonal also show agreement in simulated and fitted competitive association traces when dissociation rates are faster than 0.1 s^−1^. Accordingly, the output affinities are consistent with the true value (relative errors <0.7% and CV < 9%); however, the on‐ and off‐rates drift to extremely high values (Figure 1b), reflected in relative errors of >>500% and CV > 298% respectively. This simulation matches the experimental issue depicted in *Case 2* and will be referred to as the “fast off‐rate problem.”

### 3.2 Accurate off‐rate calculations from on‐rates and steady‐state affinities depend on sufficiently fast tracer BK and appropriate equilibration times

The results described above suggest that in the *Case 1* situation, on‐rates together with affinities from independent equilibrium endpoint experiments can indeed be used to calculate the off‐rate. To determine the minimum incubation time required for precise and accurate *K*_D,eq_ determination, we simulated our standard equilibrium probe competition assay procedure (Schiele et al., 2015) at equilibration times ranging between 1 and 24 hr and assessed the affinities of a 12‐compound subset from the 35 described above, assuming similar tracer BK (Figure S1 and Table S1, Experiment 10). The results show that steady‐state affinities are underestimated by at least a factor of 3 when incubation times are half the residence times of test compounds or shorter. On the other hand, the required incubation time will depend on the tracer BK, especially if the tracer is dissociating more slowly from the target than the test compound. Based on these analyses, it is recommended—whenever possible—to adapt incubation times of competitive equilibrium binding affinity assays to the expected test compound residence time and use the fastest binding tracer available.

### 3.3 Effects of measurement length and frequency, tracer kinetic properties, and amount of equilibrium complexes in vehicle controls on the precision and accuracy of rate constant determination

In order to gain a better understanding of the experimental factors underlying *Case 1* and *Case 2*, the subsequent analyses aimed at characterising the influence of assay parameters such as the total measurement (observation) time, the sampling frequency (measuring interval), the tracer concentration, and its BK parameters in the precision and accuracy of parameter determination by the kinetics of competitive binding model. To this end, we considered hypothetical compounds for which either the “slow off‐rate” or the “fast off‐rate problem” occurs (i.e., k_on_ [M^−1^ s^−1^], k_off_ [s^−1^]: 10^6^, 10^−4^ or 10^6^, 10^−1^ respectively) and performed further MC simulations as described in Table S1, Experiments 2 to 5.

**Figure 2:**
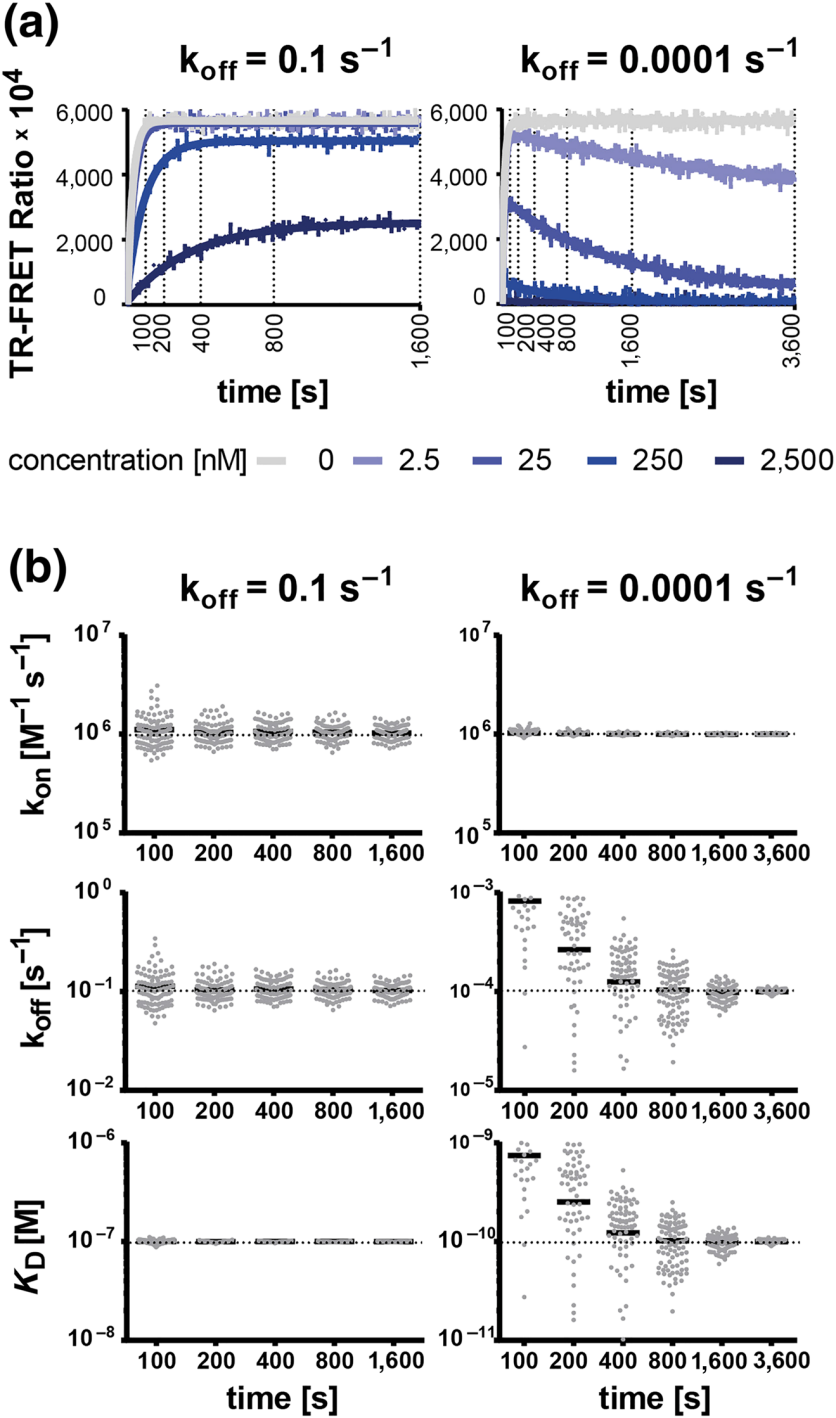
Effect of observation time on Motulsky–Mahan model fitting precision and accuracy. (a) Representative graphs of simulated competitive tracer‐compound association traces for a fast‐ (10^−1^ s^−1^) and a slow‐dissociating (10^−4^ s^−1^) compound, both with an association rate of 10^6^ M^−1^ s^−1^. The simulation assumed a measuring interval of 10 s and 12.5‐nM tracer with a k_on_ = 2.56 × 10^6^ M^−1^ s^−1^ and a k_off_ = 1.67 × 10^−3^ s^−1^. The vertical lines indicate the different total observation times used for the simulations. The corresponding fit curves derived from the “kinetics of competitive binding” equation are indicated as solid lines. (b) Monte Carlo simulations and analyses as shown in Panel (a) were performed 100 times per compound and per observation time. The graphs represent the input binding kinetic parameters (horizontal dotted line) along with the output parameters (grey dots) calculated by global fitting of the Motulsky–Mahan model to the simulated binding traces. Not all output parameters are inside the y‐axis limits. The solid black lines represent the mean values of all output parameters of a Monte Carlo experiment

The results of these experiments show that increasing the observation time from 100 to 3,600 s decreased stepwise the relative error and CV for k_off_ and *K*_D_ determinations for compounds with the “slow off‐rate problem” (k_off_ ≤ 0.0001 s^−1^) from >500% and around 150% to <4% and <22% (Figures 2, S3A, and S4A). In contrast, compounds exhibiting first signs of the “fast off‐rate problem” were less affected by the observation time, suggesting that *the total observation time determines the longest* residence time *quantifiable in “kinetics of competitive binding” experiments* and *slower off‐rates can be reliably determined by using longer incubation times*.

**Figure 3:**
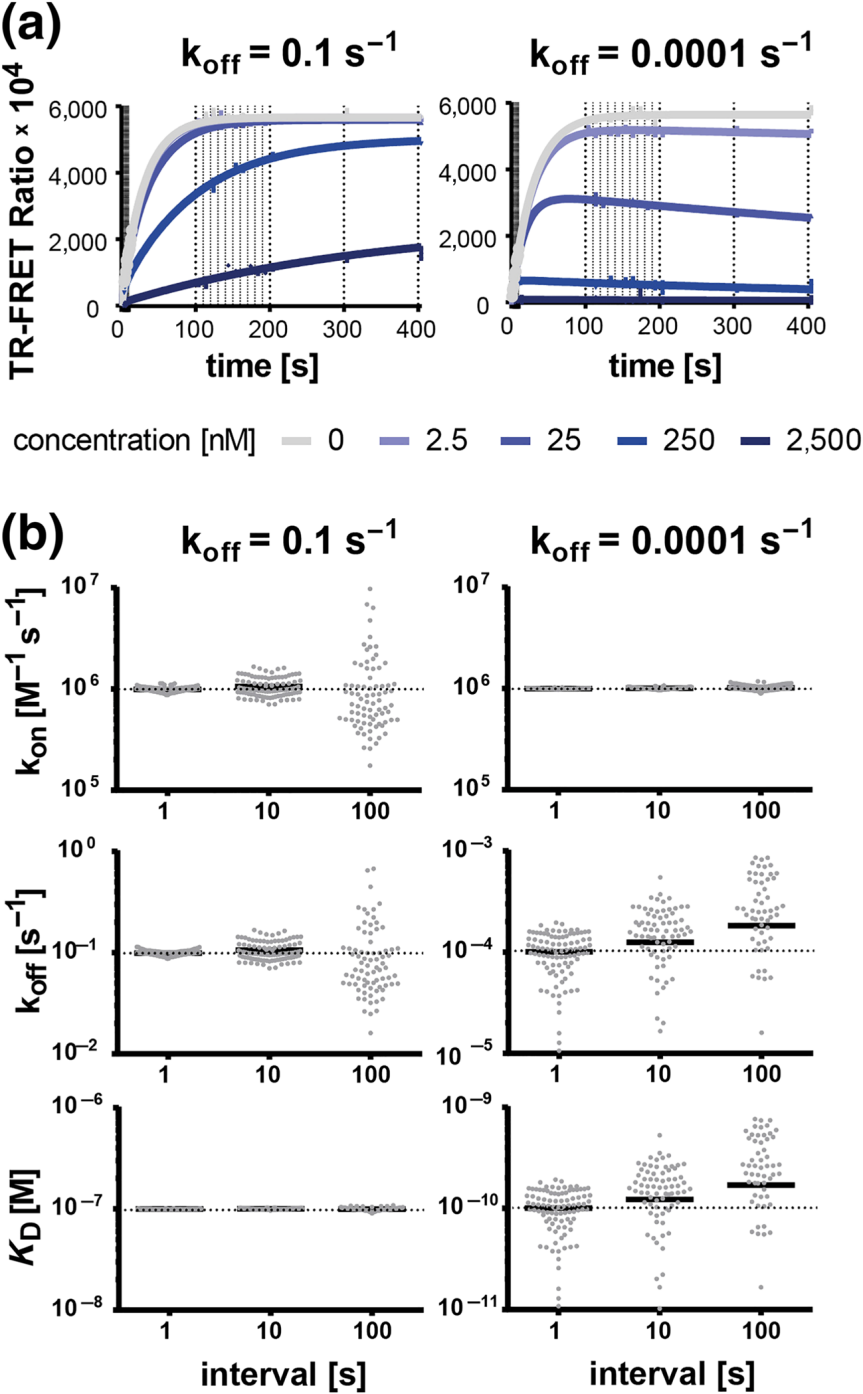
Effect of measuring interval on Motulsky–Mahan model fitting precision and accuracy. (a) Representative graphs of simulated competitive tracer‐compound association traces for a fast‐ (10^−1^ s^−1^) and a slow‐dissociating (10^−4^ s^−1^) compound, both with an association rate of 10^6^ M^−1^ s^−1^. All simulations assumed an observation time of 400 s and 12.5‐nM tracer with a k_on_ = 2.56 × 10^6^ M^−1^ s^−1^ and a k_off_ = 1.67 × 10^−3^ s^−1^. The vertical lines indicate the different measuring intervals used for the simulations. The corresponding fit curves derived from the “kinetics of competitive binding” equation are indicated as solid lines. (b) Monte Carlo simulations and analyses as shown in Panel (a) were performed 100 times per compound and per measuring interval. The graphs represent the input binding kinetic parameters (horizontal dotted line) along with the output parameters (grey dots) calculated by using the Motulsky–Mahan model. Not all output parameters are inside the y‐axis limits. The solid black lines represent the mean values of all output parameters of a Monte Carlo experiment

Decreasing the measuring interval from 100 to 1 s progressively reduced the relative error and CV for k_on_ and k_off_ determinations for “fast off‐rate problem” compounds (k_off_ ≥ 0.1 s^−1^) from >500% and >400% to around 1% and <7% (Figures 3, S3B, and S4B). Interestingly, the relative error and CV for k_off_ and *K*_D_ of the slow‐dissociating compounds (k_off_ ≤ 0.0001 s^−1^) were also affected, but the slow off‐rate problem could not be completely prevented, with CV changes from around 150% to <72%, and <20% relative error for 1‐s measuring intervals. These results strongly suggest that *measurement frequency determines the fastest dissociation rate quantifiable in “kinetics of competitive binding” experiments* and that *shorter measurement intervals enable reliable quantification of faster off‐rates*.

**Figure 4:**
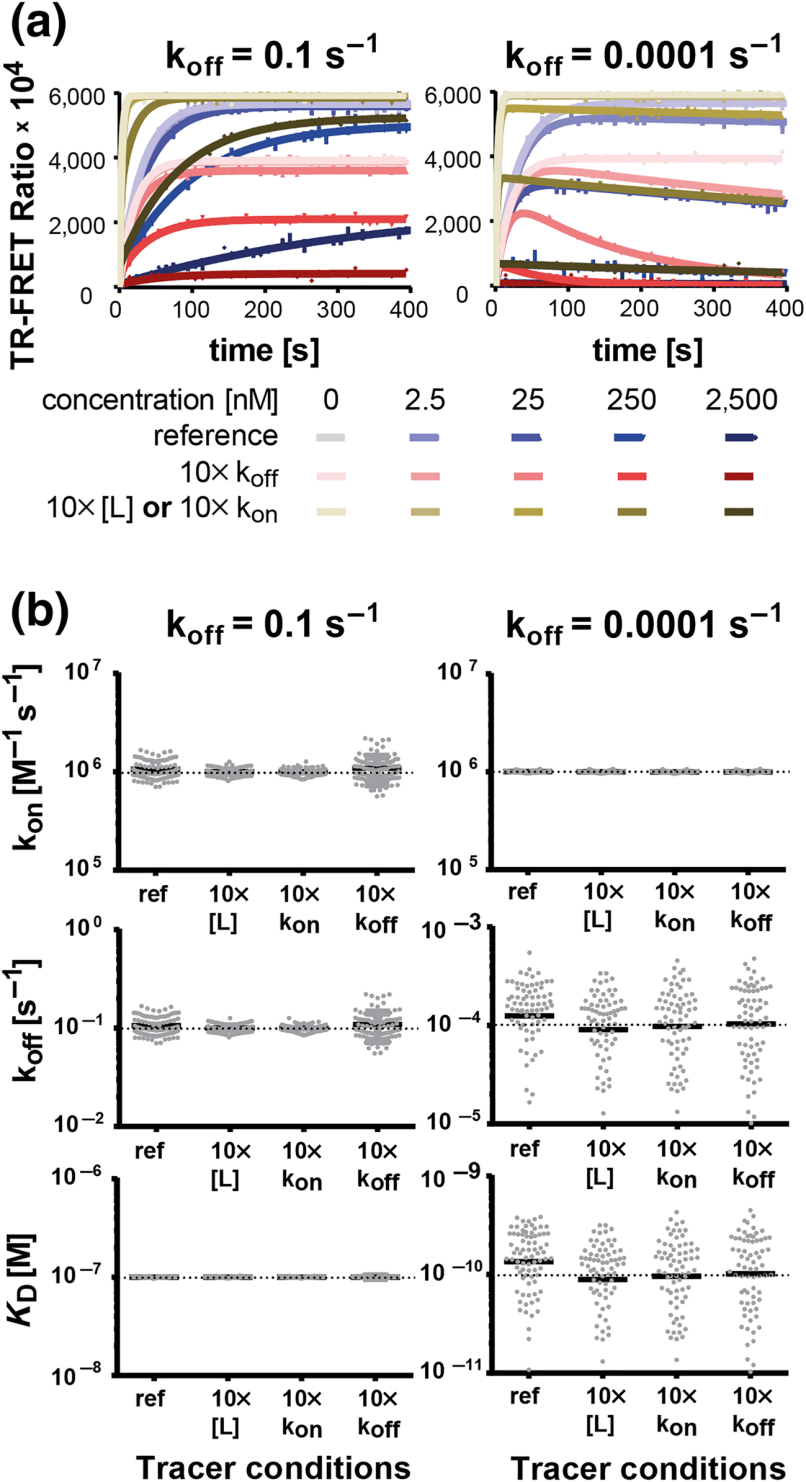
Effect of tracer binding kinetics and concentration on Motulsky–Mahan model fitting precision and accuracy. (a) Representative graphs of simulated competitive tracer‐compound association traces for a fast‐ (10^−1^ s^−1^) and a slow‐dissociating (10^−4^ s^−1^) compound, both with an association rate of 10^6^ M^−1^ s^−1^. All simulations considered an observation time of 400 s and a measuring interval of 10 s. The purple traces were simulated assuming 12.5‐nM tracer with a k_on_ = 2.56 × 10^6^ M^−1^ s^−1^ and a k_off_ = 1.67 × 10^−3^ s^−1^. The red traces considered a 10‐fold faster tracer off‐rate, while the sepia curves assumed either a 10‐fold higher tracer concentration or a 10‐fold faster on‐rate (resulting in the same traces). The corresponding fit curves derived from the “kinetics of competitive binding” equation are indicated as solid lines. (b) Monte Carlo simulations and analyses as shown in Panel (a) were performed 100 times per compound and per tracer condition. The graphs represent the input binding kinetic parameters (horizontal dotted line) along with the output parameters (grey dots) calculated by global fitting of the Motulsky–Mahan model to the simulated binding traces. Not all output parameters are inside the y‐axis limits. The solid black lines represent the mean values of all output parameters of a Monte Carlo experiment

Given the current technical limitations of increasing sampling frequency, we decided to investigate further experimental parameters influencing the “fast off‐rate problem,” such as tracer concentrations and BK properties. Figures 3C, 4, and S4C show that Motulsky–Mahan model's performance increased to a similar extent with either 10× higher tracer concentrations or 10× faster on‐rates. However, the magnitude of the improvements was smaller than those obtained by prolonged observation time or increased measuring frequency: The CVs were still >70% in the cases where the slow or fast off‐rate problem occurred. Taken together, *tracer concentration and BK have a small influence on precision and accuracy of kinetic rate constants determined in “kinetics of competitive binding” experiments* and *higher tracer concentrations or faster on‐rates have a slightly positive effect on the quantification of fast‐dissociation rates*.

Further MC analyses examining the combined effect of different assay parameters described above show that the influence of tracer association and dissociation rates on fit precision and accuracy is rather moderate (Figure S5A–D), in contrast to the marked effect of compound residence times. Consequently, changes in observation time and interval heavily influence the point at which the “slow off‐rate” and “fast off‐rate problems” become limiting (Figure S5E). Other practical considerations derived from this analysis are that (a) choosing slower dissociating tracers can slightly improve the quantification of slowly dissociating compounds (see simulations for compounds with dissociation rates of 10^−3^ s^−1^ in Figure S5A–C), (b) a tracer associating too slowly has a negative effects on the determination of fast‐dissociating compounds (see simulations for compounds with dissociation rates of 10^−1^ s^−1^ in Figure S5B and D), and (c) the relative amount of steady‐state complexes (percentage of equilibrium reached) in the vehicle control (i.e., maximum tracer occupancy) greatly influences precision and accuracy. To illustrate this last point, tracer binding with the kinetic parameters from Figure S5E can be assumed, but with a 10‐s interval for 400‐s observation time (instead of the 120‐s interval for 4,680‐s observation time used in Panel E). In this case, the tracer would reach approximately 26% instead of 97% equilibrium and no BK parameters could be quantified for the test compounds (not shown). Finally, comparing the output parameters of Figure S5A,C,D reveals that the upper limits for on‐rate determination depend on tracer affinity and concentration. These limits can be pushed by using lower compound concentrations or higher tracer concentrations (not shown).

### 3.4 Characteristic signatures of experimental and conceptual mistakes affecting the precision and accuracy of rate constant determination

The performance of parameter fitting by the kinetics of competitive binding model can be affected by experimental errors and unsuitable interaction mechanisms (i.e., distinct from the simple 1:1 reversible binding assumed by the equation). In order to assess to what extent these mistakes affect the signal traces and the accuracy of BK quantification, competition association traces were simulated under assumption of either (a) input values for compound concentration, tracer concentration, or tracer BK different from those used for the subsequent evaluation with the Motulsky–Mahan equation or (b) more complex interaction mechanisms (details in Table S1). To this end, we considered the analysis of both a slow‐ and a fast‐dissociating hypothetical compound with an assay set‐up allowing accurate and precise parameter determination (k_on_ [M^−1^ s^−1^], k_off_ [s^−1^], observation time [s], and interval [s]: 10^6^, 10^−4^, 1,600, 10 and 10^6^, 10^−2^, 400, 10). Our simulations revealed that most errors are reflected by specific patterns (or differences between the signal traces and the corresponding fit curves), which can be robustly detected when the magnitude of the errors in compound concentration surpass 40% (Figures S6–S7). In agreement with our observation for slowly dissociating compounds, the on‐rates for an irreversible binding compound could be also quantified with high accuracy (Figure S7B). Interestingly, deviations from the true tracer concentration and BK, as well as compound concentration errors in a single well, accounted for the biggest accuracy issues (Figure S7A), with different dependencies of effect, direction, and magnitude for fast‐ and slow‐dissociating compounds.

### 3.5 Simultaneous determination of association and dissociation kinetics in a single titration experiment favours precision and accuracy of tracer characterisation

**Figure 5:**
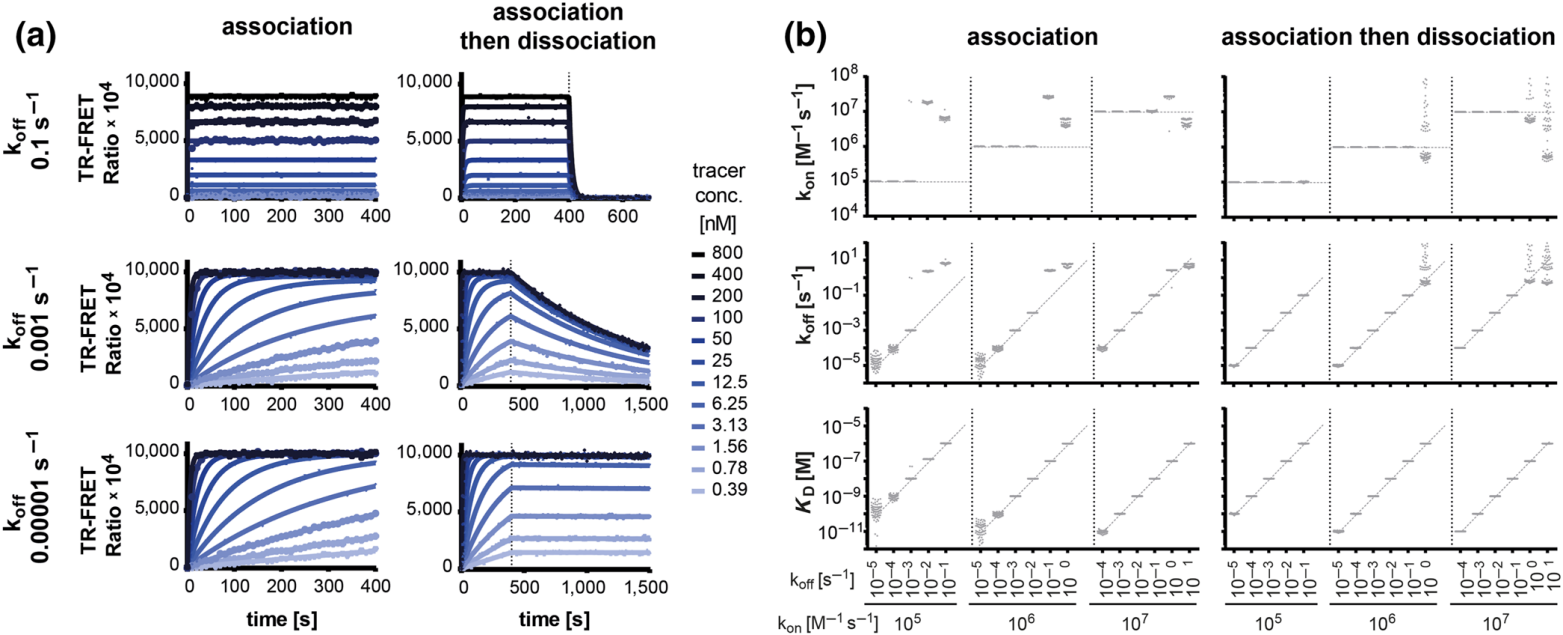
Comparison of two experimental approaches for tracer characterisation. (a) Representative graphs of simulated tracer association traces as well as tracer association‐then‐dissociation traces for tracers with an association rate of 10^6^ M^−1^ s^−1^ and with different dissociation rates (0.1 vs. 0.001 vs. 0.00001 s^−1^). The solid lines represent the corresponding fit curves. (b) Monte Carlo simulations and analyses similarly or identical to those shown in (a) were performed 100 times respectively. The graphs represent the input binding kinetic parameters (horizontal and diagonal dashed lines) along with the output parameters (grey dots) calculated by using either the “association kinetics” or the “association‐then‐dissociation” model. Not all output parameters are inside the y‐axis limits

Tracer binding parameters are commonly determined in kinetic association experiments followed by data fitting to a pseudo‐first‐order exponential rate equation. Sometimes, a displacement (chase) experiment is performed separately, and the off‐rate can be determined by fitting the curve to a single phase exponential decay model (Schiele et al., 2015). Recently, we described an alternative approach where binding of increasing tracer doses is followed by displacement by an excess of “cold” tracer within the same experiment, and the kinetic traces are globally fitted to an ‘*association‐then‐dissociation*’ model accounting for various tracer concentrations (de Witte et al., 2018; Nederpelt et al., 2016). In order to compare the precision and accuracy of these methods, we conducted MC simulations for a series of hypothetical tracers spanning a broad range of on‐ and off‐rates (examples are shown in Figure 5a) and included a random fluctuation of the fluorescence signals. Data were then evaluated with the respective multivariable non‐linear models (Table S1, Experiments 8 and 9) and fit performances were compared. Despite similar overall performance, the analysis suggests that the approach combining association and dissociation is beneficial when dealing with fast‐dissociating tracers (Figure 5b). “Kinetic association assays” showed lower precision and accuracy for determination of slowly dissociating compounds (10^−5^ s^−1^), an issue that can be prevented by longer incubation times (not shown). For tracers with low affinities (<10^−7^ M) and fast‐dissociation rates, the corresponding kinetic rate constants could not be determined accurately. With the “kinetic association‐then‐dissociation assay,” all off‐rates <1 s^−1^ (and the corresponding on‐rates) could be determined with high precision and accuracy, regardless of the affinity of the tracer, and performance issues were only observed for faster dissociating probes. Moreover, the association‐then‐dissociation signal traces can be interpreted more intuitively, allowing for easy recognition of slow‐ and fast‐dissociating tracers (Figure 5a).

### 3.6 Systematic fluorescence signal decay can be addressed both analytically and with raw data normalisation approaches

**Figure 6:**
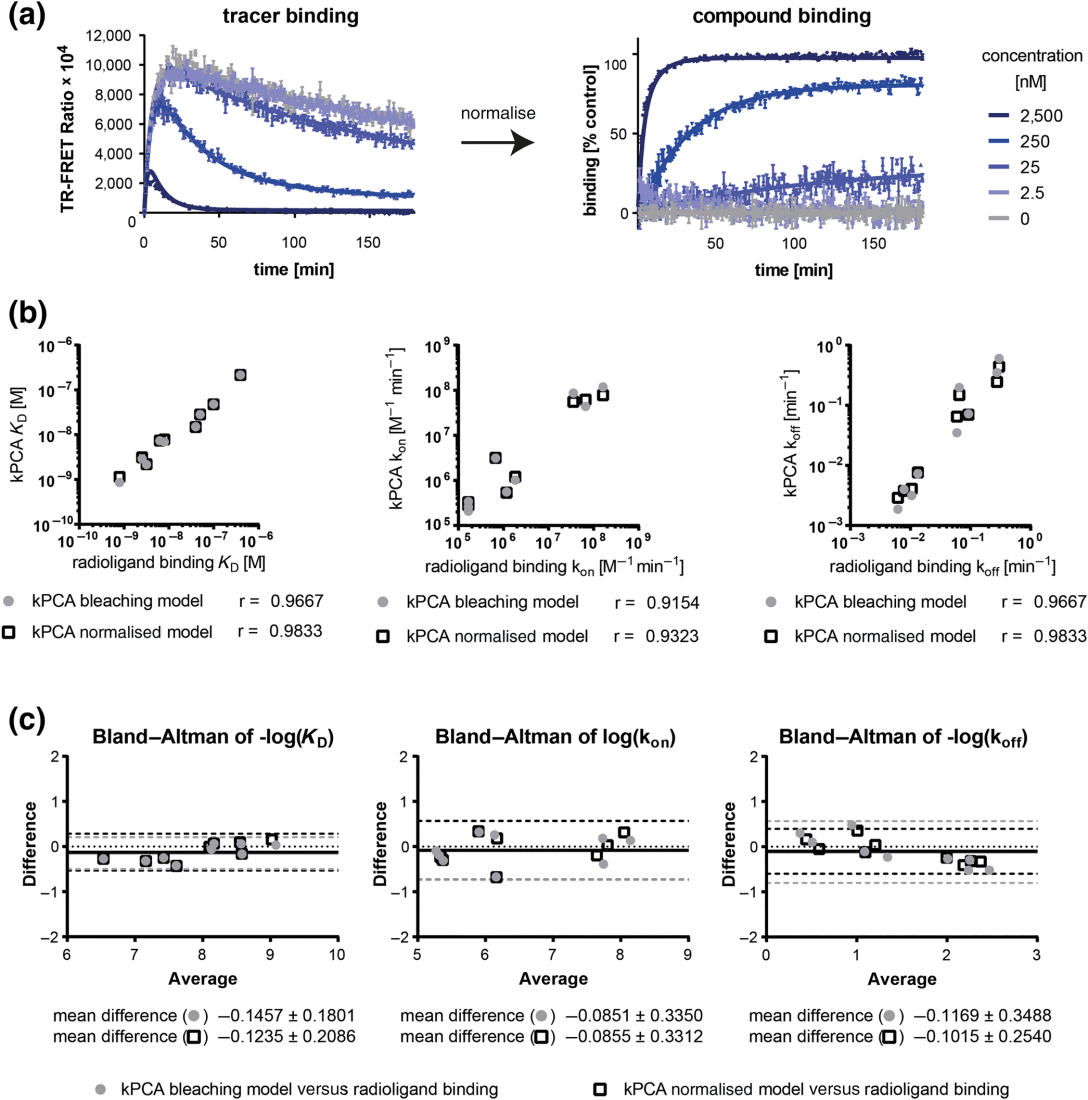
Comparison of two kinetics of competitive binding models dealing with systematic signal decay. (a) The left graph shows an example of a competitive tracer‐compound binding trace with systematic signal decay (from kPCA experiment, Bosma et al., 2019) and the corresponding fit (solid line) derived from the Motulsky–Mahan model equation multiplied with a signal drift term where *K*_Drift_ = 0.0028 ± 0.0004. The signal is also decaying in the control well without compound. The right graph shows the associated normalised traces: The percentage of binding was calculated by normalisation between the tracer binding control (0% compound binding) and the background signal (100% compound binding). The corresponding fit (solid line) was calculated by using the normalised Motulsky–Mahan equation. (b–c) For nine compounds, the resulting binding parameters from both fits were compared to those obtained from radioligand binding experiments (Bosma et al., 2019) represents the correlation plots. Spearman correlation coefficients are indicated below the graphs. Panel (c) shows Bland–Altman plots. The solid horizontal lines indicate the mean log difference for all data points—which is also given as mean ± SD below the graphs. The dashed lines represent the upper and lower 95% limit of agreement

Fluorescence‐based competition association assays are sometimes affected by signal decay over time, which is not attributable to the pharmacological events being investigated (Bosma et al., 2019; Nederpelt et al., 2016; Schiele et al., 2015). We previously hypothesised this loss of fluorescence to be linked to photobleaching and introduced an equation in which the Motulsky–Mahan model was extended to account for this phenomenon (Schiele et al., 2015). With further expansion of our assay portfolio and expertise, we have noticed that fluorescence losses also depend on the nature of the target, the background, buffer and tracer warhead. Therefore, here, we developed a more general approach consisting on a modified Motulsky–Mahan model that can be used to fit normalised kinetic traces. Figure 6a shows that both correction procedures generate better fits to literature data for which comparative radioligand experiments with no signal drift were available (Bosma et al., 2019). Parameters (k_on_, k_off_, and *K*_D_), obtained with both models, are in good agreement with data from orthogonal assays (*r* > 0.92, mean log difference < 0.15, Figure 6b–c). Nevertheless, the correlation is slightly better for normalised fluorescence data (mean log difference: 0.10 ± 0.25 vs. 0.12 ± 0.35 for photobleaching model). Both methods are now available in the Genedata Screener® software,(Dubrovskiy et al., 2016) and can be easily implemented in comparable analysis software.

## 4 DISCUSSION AND CONCLUSIONS

Differences in the clinical outcomes of drugs addressing a particular medical need are often defined by their molecular mode of action and—more specifically—by their target interaction kinetics (Swinney, 2004; Swinney, 2006). Not surprisingly, this parameter is nowadays monitored in drug discovery programmes all the way from the hit identification and lead optimisation phases up to the candidate evaluation and selection (Georgi et al., 2017). To this end, the Motulsky–Mahan “kinetics of competitive binding” model is widely applied, with recent efforts aiming at screening assay formats with increased robustness and throughput (Antoine et al., 2016; Guo et al., 2012; Schiele et al., 2015; Stoddard et al., 2018).

In this study, we have addressed challenges to the Motulsky–Mahan model's performance observed under typical experimental conditions of drug screening laboratories (i.e., considering the technical limitations imposed by state‐of‐the‐art assay technologies and instrumentation), with the aim of providing experimenters with simple diagnostic tools to identify concrete issues we have encountered in our daily practice. To this end, we used the timescale and compound doses typically used in our laboratory, nonetheless the mathematical background and results derived from the analyses will be identical if the parameters are changed. We also assumed—based on experience—a rather low‐signal fluctuation, equal for all compound concentrations tested. Consequently, the quantitative outcome from MC analyses performed with other fluctuation levels will be different, while the qualitative conclusions should remain unchanged. An alternative approach to ours would have been to use the principles inherent in the equation to design an appropriate range and spacing (probably geometric) of binding measurements so that the rate constants of the competing ligands can be estimated using regression analysis. Knowing the kinetics of the tracer, we could describe how it is affected by competitors with different rate constants. This strategy would also help design and verify appropriate time points to measure the rate constants of competitors, although the recommendations derived from it might not always be practicable.

The most important finding of our study is the quantification of the magnitude at which the precision and accuracy of the Motulsky–Mahan model are altered in “real life” conditions by (a) compound dissociation rates, (b) measurement frequency, and (c) total observation time, with the last two elements dictating the threshold for the limitations imposed by the first. Based on our observations, we can propose a few “rules of thumb” for the design, analysis, and interpretation of competition association experiments:

- When the residence time of the compound is fivefold longer than the observation time, neither k_4_ nor *K*_D_ can be precisely fitted. In contrast, k_3_ is determined with high precision and accuracy. While observation time can be increased to circumvent this problem; this solution decreases the throughput and will be often limited by the stability of assay components. Alternatively, off‐rates can be calculated by multiplying the precise Motulsky–Mahan on‐rate and the affinity determined in an independent equilibrium assay (k_off_ = *K*_D_ × k_on_). In this context, attention should be paid to the fact that the calculated steady‐state affinity can be influenced by the incubation time, as well as by ligand and target depletion (assay wall) issues (Aranyi, 1980; Easson & Stedman, 1936; Heise, Sullivan, & Crowe, 2007; Klebl, Müller, & Hamacher, 2011). Looking for discrepancies when comparing *K*_D,eq_ and *K*_D,kin_ as recommended in the GraphPad Curve Fitting Guide (Motulsky, 1995 GraphPad Software, Inc.) is a helpful exercise to assess whether this approach is justified. Future developments of the model could include global fitting of competitive association (kPCA) traces and steady‐state competition (ePCA) curves..
- If k_4_ is faster than the measurement frequency, neither k_3_ nor k_4_ can be correctly fitted, but the affinity is still determined with utmost precision and accuracy. This feature can be used as empirical tool to identify fast‐dissociating compounds. Importantly, this implies that a good correlation of *K*_D,eq_ and *K*_D,kin_ is not a sufficient quality criterion for k_3_ and k_4_ determinations. According to the GraphPad Curve Fitting Guide, the Motulsky–Mahan model only gives reliable results if there are many measuring signals in early time points (Motulsky, 1995 GraphPad Software, Inc.). Our results corroborate this recommendation for fast‐dissociating compounds but also show that early time points are less relevant for slow‐dissociating compounds. Of note, covering the signal increase phase with many data points might not be sufficient to quantify fast off‐rates. In fact, the highest k_4_ that can be measured is dictated by the length of the first sampling interval, which is often limited in current instrumentation. These findings raise a warning regarding the “kinetic rate index” recently introduced by Guo et al. (2013): Fast‐dissociating compounds cannot be ranked by their off‐rate using this approach, as it does not consider time points before tracer equilibration in the vehicle control. Furthermore, the choice of the second measurement time point in Guo's method will determine its ability to rank slow k_4_ compounds.
- The lower and upper limits for measurable k_3_ values are determined by the interplay between tracer and compound affinity and concentration. Thus, fit precision and accuracy issues can be improved by increasing the dynamic window of the assay with adjusted tracer and/or compound doses. Fast‐associating tracers, or alternatively higher tracer concentrations, improve the threshold for the fastest k_4_ that can be reliably determined, and slow‐dissociating tracers enhance the precision for quantitation of slow off‐rate compounds. In contrast, slow k_1_ tracers produce lower signal‐to‐noise ratios in early time points, with a consequent negative impact on fit quality.

Some of these findings, especially the observation that the certainty of BK determination depends on the off‐rate of the compound, have been recently discussed in a study comparing various tracers and readout technologies (Bosma et al., 2019). In that paper, the choice of slow‐ and fast‐dissociating tracers was suggested to be the main factor influencing experimental outcomes. Here, we provide an additional explanation to the differences observed, relying on the distinct measuring intervals and observation times chosen. Along these lines, here, we remind readers that k_1_ and k_2_ accuracies are critical for the quality of k_3_ and k_4_ determinations and demonstrate that experimental tracer characterisation approaches involving “association‐then‐dissociation of multiple tracer concentrations” can increase the accuracy of parameter determination. Also in relation to (Bosma et al., 2019), where the HTRF‐based assay was affected by a systematic signal drift, here, we propose that normalisation of the Motulsky–Mahan model and signal traces is a valuable alternative to cope with this issue, especially in cases where photobleaching might not be the only source of fluorescence decay or the latter is not mono‐exponential. Of note, photobleaching is often more complex than a mono‐exponential process (Rigaut & Vassy, 1991; Song, Hennink, Young, & Tanke, 1995), and the signal decrease can be related to other phenomenon such as cell sedimentation or protein target degradation. Having said this, the downside of a normalised model with no parameter for B_max_ is the potentiation of issues associated with noisy signal traces and fast‐dissociating compounds.

In conclusion, this study provides new insights into the dependence of the Motusky–Mahan model on experimental parameters. Experimentalists are encouraged to use the MC simulations presented here for detailed evaluation of their own procedures (adjustments may be necessary if they differ significantly from the kPCA assay set‐up, Schiele et al., 2015) and to address the remaining open questions, such as the influence of ligand and target depletion. Along these lines, further studies could include the expansion of these analyses to recent variants of the “kinetics of competitive binding” equation that incorporate alternative scheduling of the labelled and unlabelled ligand addition (Hoare, 2018; Shimizu, Ogawa, & Nakayama, 2016) or two‐state binding (Guo et al., 2018). All in all, this work should enable the optimised design and analysis of competition association assays.

## AUTHOR CONTRIBUTIONS

V.G. and A.F.M. initiated the project. V.G. designed the study and performed the analyses under supervision of A.F.M. V.G., A.D., S.S., and A.F.M. contributed to deriving model equations. V.G. wrote the paper under supervision of and input from A.F.M. and with input from A.D. and S.S.

## CONFLICT OF INTEREST

S.S. is employed by Genedata AG, Switzerland.

## DECLARATION OF TRANSPARENCY AND SCIENTIFIC RIGOUR

This Declaration acknowledges that this paper adheres to the principles for transparent reporting and scientific rigour of preclinical research as stated in the *BJP* guidelines for https://bpspubs.onlinelibrary.wiley.com/doi/abs/10.1111/bph.14207, and as recommended by funding agencies, publishers and other organisations engaged with supporting research.

## Supporting information


**Table S1** Summary of Monte Carlo (MC) analyses and simulation experiments performed in this study.
**Figure S1** Time course of apparent affinities calculated from simulated equilibrium probe competition assays (ePCA) depending on compounds binding kinetics. ePCA experiments were simulated for 12 hypothetical compounds with different on‐ and off‐rates. All simulations considered 12.5 nM tracer with a kon = 2.56 × 106 M‐1 s‐1 and a koff = 1.67 × 10–3 s‐1. The graph shows the apparent affinities (y‐axis) derived from the obtained dose–response curves after different incubation times (x‐axis). Data points given with the same color or symbol come from compounds with the same dissociation rate or association rate, respectively.
**Figure S2** Simulated time courses of competitive tracer‐compound association traces illustrating the slow or the fast off‐rate problem. The simulation considered an observation time of 400 s, a measuring interval of 10 s, and 12.5 nM tracer with a kon = 2.56 × 106 M‐1 s‐1 and a koff = 1.67 × 10–3 s‐1. (A) Simulated traces for compounds with the same on‐rate, but different off‐rates. The lower graphs for compounds with dissociation rates of 10–4 s‐1 or slower show the same binding profile. (B) Simulated traces for compounds with the same affinity, but different on‐ and off‐rates. The upper two graphs for compounds with dissociation rates of 10–1 s‐1 or faster show similar binding profiles. The top plots zoom into the first 40 seconds of the graphs below. The earliest time point (first 10 s interval) of measurement of the ‘250 nM compound trace’ is higher for the compound with a dissociation rate of 10–1 s‐1 than for those with a faster dissociation rate.
**Figure S3** Simulated time courses of competitive tracer‐compound association traces illustrating how to reduce the slow or the fast off‐rate problem. If not otherwise specified, the simulations considered an observation time of 400 s, a measuring interval of 10 s, and 12.5 nM tracer with a kon = 2.56 × 106 M‐1 s‐1 and a koff = 1.67 × 10–3 s‐1. (A) Simulated traces with an increased observation time of 1 h for compounds with the same on‐rate, but different off‐rates. The arrows indicate the decreasing area under the curve of the ‘250 nM compound trace’ with slower dissociation rates, which can be observed at the end of the observation period. (B) Simulated traces with a faster measuring interval of 1 s for compounds with the same affinity, but different on‐ and off‐rates. The bottom plots zoom into the first 10 seconds of the graphs above. At the early time points (1–10 s) of measurement the area under curves are larger for the compound with a dissociation rate of 10–1 s‐1 than for those with a faster dissociation rate. (C) Simulated traces with different tracer binding kinetics (as indicated above the graphs) for compounds with the same affinity, but different on‐ and off‐rates. The arrows indicate the higher signals at the earliest time point (first 10 s interval) of measurement with decreasing dissociation rate of the compound.
**Figure S4** Precision (CV) and accuracy (relative error) of the Motulsky‐Mahan model depending on observation time (A), measuring interval (B) and tracer characteristics (C). If not otherwise specified, the simulations considered an observation time of 400 s, a measuring interval of 10 s, and 12.5 nM tracer with a kon = 2.56 × 106 M‐1 s‐1 and a koff = 1.67 × 10–3 s‐1. Panel A, B and C show the results from the analysis in Figures 2B, 3B and 4B, respectively, where the simulations were performed for a fast (10–1 s‐1) and a slower dissociating (10–4 s‐1) compound, both with an association rate of 106 M‐1 s‐1 (grey). Additionally, panel A, B and C present the result for the same analysis, but with an alternative association rate of 108 M‐1 s‐1 (green). Moreover, panel C depicts CVs and the relative errors determined from a similar Monte Carlo analysis, but for a faster dissociating (1 s‐1) compound with an association rate of 108 M‐1 s‐1 (purple).
**Figure S5** Performance of the Motulsky‐Mahan model depending on tracer and compound binding kinetics, tracer concentration, incubation time and measuring interval. Similar to the analysis in Figure 1, Monte Carlo simulations and analyses (100 simulations of kinetic probe competition experiments, respectively) were performed for 35 hypothetical compounds with different on‐ and off‐rates. The simulations considered an observation time t, a measuring interval I, a tracer concentration c, and tracer binding kinetics (kon, koff) as indicated above the rate plots in panel A‐E. The rate plots represent the input binding kinetic parameters (large symbols) for the 35 compounds (one color per compound) as used for the simulation along with the corresponding output rates (small symbols) calculated by using the Motulsky‐Mahan model. The diagonals in the plots are isoaffinity lines. All plots zoom into the range of the input parameters. Thus, not all output parameters are inside the axis limits.
**Figure S6** Effect of experimental errors on simulated time courses of competitive tracer‐compound association traces and the corresponding global fit of the Motusky‐Mahan equation to the data points.
**Figure S7** Effect of experimental and conceptual errors on the results of kinetics of competitive binding experiments. (A) The effect of experimental errors on accuracy. Monte Carlo simulations and analyses similarly or identical to those shown in Supplementary Figure 6 were performed 100 times, respectively. The graphs represent the input binding kinetic parameters (horizontal dashed line) along with the output parameters (grey dots) calculated by using the Motulsky‐Mahan model. Not all output parameters are inside the y‐axis limits. (B) The effect of conceptual errors on simulated time courses of competitive tracer‐compound association traces and the corresponding global fit of the Motusky‐Mahan equation to the data points. The simulations considered an observation time of 400 s, a measuring interval of 10 s, 12.5 nM tracer with a kon = 2.56 × 106 M‐1 s‐1 and a koff = 1.67 × 10–3 s‐1, and a compound binding irreversible or via induced fit to the target molecule. However, the model equation used for the analysis assumes a simple 1:1 binding model. The arrows indicate the deviations between the best‐fit curves and the corresponding signal trace.

Data S2. Supporting Information.

Data S3. Supporting Information.

Data S4. Supporting Information.

Data S5. Supporting Information.
