# Supplementary material for: Considerations for improved performance of competition association assays analysed with the Motulsky–Mahan's “kinetics of competitive binding” model
Source: Br J Pharmacol. 2019 Dec 26;176(24):4731–44. doi: 10.1111/bph.14841 (PMC7029771; doi:10.1111/bph.14841)
Supplement: Supplementary file 5 — Data S5. Supporting Information. [file BPH-176-4731-s001.pdf]

Supplementary information

Supplementary methods

Models and equations.

Motulsky-Mahan 'kinetics of competitive binding' model (GraphPad Prism equation):

$$Y(t) = \frac{B_{max} \times k_1 \times L}{Diff}\left(\frac{k_4\ Diff}{K_F K_S} + \frac{k_4 - K_F}{K_F}\ e^{-K_F t} - \frac{k_4 - K_S}{K_S}\ e^{-K_S t}\right)$$

with

$K_A = k_1 \times L + k_2$

$K_B = k_3 \times I + k_4$

$K_F = 0.5\ (\ K_A + K_B + \sqrt{(K_A - K_B)^2 + 4 \times k1 \times k3 \times L \times I}\ )$

$K_S = 0.5\ (\ K_A + K_B - \sqrt{(K_A - K_B)^2 + 4 \times k1 \times k3 \times L \times I}\ )$

$Diff = K_F - K_S$

$K_D = k_4 / k_3$

where: Y: specific tracer binding signal [arbitrary binding unit]; t: time [s]; k_1, k_2, L: association rate [$M^{-1}s^{-1}$], dissociation rate [s^{-1}] and concentration [M] of the tracer, k_3, I, k_4: association rate [$M^{-1}s^{-1}$], concentration [M] and dissociation rate [s^{-1}] of unlabelled compound; B_{max}: maximum binding signal at very high concentration of tracer (often exceeds what is measured in the experiment).

'kinetics of competitive binding' equation with signal drift term (Schiele et al., 2015),

The Motulsky-Mahan 'kinetics of competitive binding' equation Y(t) is multiplied by a mono-exponentially decreasing signal drift term

$$Y_{Drift}(t) = Y(t) \times e^{-k_{Drift} \times t}$$

where: Y(t): specific tracer binding signal; t: time; $Y_{Drift}(t)$: specific tracer binding signal with systematic signal decay; k_{Drift}: rate of mono-exponential decay.

1:1 irreversible compound:target interaction with 1:1 reversible tracer binding:

Biochemical model:

Target T + Compound C $\overset{k_{on}}{\longrightarrow}$ Target-Compound Complex TC

Target T + Tracer L $\underset{k_d}{\overset{k_a}{\rightleftarrows}}$ Target-Tracer Complex TL

Mathematical model:

1) $\frac{d([C](t))}{dt} = -k_{on} \times [C](t) \times [T](t)$

2) $\frac{d([TC](t))}{dt} = k_{on} \times [C](t) \times [T](t)$

3) $\frac{d([T](t))}{dt} = k_d \times [TL](t) - k_a \times [L](t) \times [T](t) - k_{on} \times [C](t) \times [T](t)$

4) $\frac{d([L](t))}{dt} = k_d \times [TL](t) - k_a \times [L](t) \times [T](t)$

5) $\frac{d([TL](t))}{dt} = k_a \times [L](t) \times [T](t) - k_d \times [TL](t)$

where: t: time; [C], [T], [TC], [L], [TL]: concentration of free compound, free target, compound-complexed target, free tracer and tracer-complexed target, respectively; k_{on}: rate constant for association of the target-compound complex; k_a, k_d: rate constants for association and dissociation of the target-tracer complex.

Compound induced fit with 1:1 reversible tracer binding

Biochemical model:

Target T + Compound C $\underset{k_{off}}{\overset{k_{on}}{\rightleftarrows}}$ intermediate Target-Compound Complex TC $\underset{k_c}{\overset{k_i}{\rightleftarrows}}$ induced Target-Compound Complex T*C

Target T + Tracer L $\underset{k_d}{\overset{k_a}{\rightleftarrows}}$ Target-Tracer Complex TL

Mathematical model:

1) $\frac{d([C](t))}{dt} = k_{off} \times [TC](t) - k_{on} \times [C](t) \times [T](t)$

2) $\frac{d([TC](t))}{dt} = k_{on} \times [C](t) \times [T](t) - k_{off} \times [TC](t) - k_i \times [TC](t) + k_c \times [T^*C](t)$

3) $\frac{d([T^*C](t))}{dt} = k_i \times [TC](t) - k_c \times [T^*C](t)$

4) $\frac{d([T](t))}{dt} = k_{off} \times [TC](t) - k_{on} \times [C](t) \times [T](t) + k_d \times [TL](t) - k_a \times [L](t) \times [T](t)$

5) $\frac{d([L](t))}{dt} = k_d \times [TL](t) - k_a \times [L](t) \times [T](t)$

6) $\frac{d([TL](t))}{dt} = k_a \times [L](t) \times [T](t) - k_d \times [TL](t)$

where: t: time; [C], [T], [TC], [T*C], [L], [TL]: concentration of free compound, free target, transient intermediate of compound-complexed target, conformationally changed target-compound complex, free tracer and tracer-complexed target, respectively; k_{on}, k_{off}: rate constants for association and dissociation of the intermediate target-compound complex; k_i, k_c: rate constants for TC-T*C interconversion; k_a, k_d: rate constants for association and dissociation of the target-tracer complex.

'association kinetics – two or more concentrations of hot' (GraphPad Prism equation):

$K_D = k_{off} / k_{on}$

$k_{obs} = k_{on} * L + k_{off}$

Occupancy = L / (L + K_D)

Y_{max} = Occupancy * B_{max}

$Y = Y_{max} * (1 - \exp(-1 * k_{obs} * X))$

where: Y: specific tracer binding signal [arbitrary binding unit]; X: time [s]; K_D: calculated equilibrium dissociation constant [M], k_{obs}: observed rate of tracer-target complex formation, k_{off}, k_{on}, L: association rate [$M^{-1}s^{-1}$], dissociation rate [s^{-1}] and concentration [M] of the tracer; Y_{max}: maximum observable signal at equilibrium; B_{max}: maximum binding signal at very high tracer concentrations

'association-then-dissociation – two or more concentrations of hot' (Nederpelt *et al.*, 2016; de Witte *et al.*, 2018)

$K_D = k_{off} / k_{on}$

$k_{obs} = k_{on} * L + k_{off}$

Occupancy = L / (L + K_D)

Y_{max} = Occupancy * B_{max}

Association = $Y_{max} * (1 - \exp(-1 * k_{obs} * X))$

$Y_{Time0} = Y_{max} * (1 - \exp(-1 * k_{obs} * Time0))$

Dissociation = $Y_{Time0} * \exp(-1 * k_{off} * (X - Time0))$

Y = IF (X < Time0, Association, Dissociation) + NS

where: Y: specific tracer binding signal [arbitrary binding unit]; X: time [s]; K_D: calculated equilibrium dissociation constant [M], k_{obs}: observed rate of tracer-target complex formation, k_{off}, k_{on}, L: association rate [$M^{-1}s^{-1}$], dissociation rate [s^{-1}] and concentration [M] of the tracer; Y_{max}: maximum observable signal at equilibrium; B_{max}: maximum binding signal at very high tracer concentrations; Time0: time at which dissociation is initiated (e.g. by adding high concentration of unlabelled competitor); Y_{Time0}: binding signal at Time0; NS: non-specific binding signal (was constrained to 0, as specific binding signal was used)

'Three-parameter sigmoidal dose-response curve' model with a Hill slope of -1 (GraphPad Prism equation):

$$Y = Bottom + (Top - Bottom) / (1 + 10^{(X - Log(IC_{50}))})$$

where: Y: normalised tracer binding signal (response); Bottom: bottom plateau (minimal response in background control); Top: maximum response (tracer binding without compound control); X: logarithms of compound concentrations as used in the simulation; IC_{50}: concentration of compound required to provoke a response half way between Bottom and Top [in units of compound concentration].

Apparent IC_{50} values were then converted into apparent $K_{D,eq}$ values with the help of the Cheng-Prusoff equation (Cheng *et al.*, 1973):

$$K_{D,eq} = IC_{50} / \left(1 + \frac{[L]}{K_{D_L}}\right)$$

where: $K_{D,eq}$: equilibrium dissociation constant of compound; IC_{50}: concentration of compound required to provoke a response half way between minimum and maximum response; L, K_{D_L}: concentration and equilibrium dissociation constant of the tracer as used in the simulation.

Supplementary Table S1

Summary of Monte Carlo (MC) analyses and simulation experiments performed in this study.

**Experiment No.**	**1**	**2**	**3**	**4**	**5**	**6**	**7**	**8**	**9**	**10**
**Scope**	Evaluate precision and accuracy of the Motulsky-Mahan model to analyze typical assay data	Evaluate impact of observation time in model performance	Evaluate impact of measurement intervals in model performance	Evaluate impact of different tracer BKs or concentrations in model performance	Evaluate impact of various changed parameters, such as tracer BK and concentration as well as observation time and interval in model performance	Evaluate impact of experimental errors in model performance	Evaluate impact of unsuitable binding mechanisms in model performance	Comparison of models describing the kinetics of tracer-target binding		Evaluate impact of incubation time on the determination of affinity constants
**Software**	GraphPad Prism						COPASI	GraphPad Prism		
**Model for simulation**	'kinetics of competitive binding' (Motulsky-Mahan)						'1:1 irreversible interaction' or 'induced fit'	'association kinetics – two or more concentrations of hot'	'association-then-dissociation – two or more concentrations of hot'	'kinetics of competitive binding' (Motulsky-Mahan)
**Model for evaluation**	'kinetics of competitive binding' (Motulsky-Mahan)							'association kinetics – two or more concentrations of hot'	'association-then-dissociation – two or more concentrations of hot':	'Three-parameter sigmoidal dose-response curve' and Cheng-Prusoff equation (Cheng et al., 1973)
**Emulated Assay Type**	Competition association assays (kPCA in Schiele et al., 2015)							Tracer association kinetics	Tracer association and dissociation ('chase') kinetics	Equilibrium probe competition assays (ePCA in Schiele et al., 2015)
**# of Samples (compound & tracer combinations)**	35 compounds (MC analyses Fig 1; some simulation examples in Fig S2)	3 (simulation Fig. S3A) and 4 (MC analyses Fig 2 & S4)	3 (simulation Fig. S3B) and 4 (MC analyses Fig 3 & S4)	15 (simulation Fig. S3C) and 5 (MC analyses Fig 4 & S4)	35 compounds per tracer condition (MC analyses in Fig S5)	2 compounds per error (MC analyses Fig S7A; simulation examples in Fig S6)	3 compounds (simulation in Fig S7B)	17 tracer (MC analyses Fig 5)		12 compounds (Figure S1)
**N (# of conc.)**	6							10		15
**n (replicates)**	2						-	2		-
**[Compound]**	4-point 10-fold dilution series starting from 2.5 × 10^6 M), and 1000 M for background signal (to simulate 0% tracer binding) and 0 M as tracer binding control					As in Exp. No. 1 or simulation of error *1-3	As in Exp. No. 1	-		13-point 3.5-fold dilution series starting from 2 × 10^5 M); and 10 M for background signal; 0 nM as tracer binding control
**Compound's kinetic and / or affinity parameters**	Figure 1A, S2	Figures 2, S3A, S4	Figure 3, S3B, S4	Figure 4, S3C, S4	Figure S5	Figures S6, S7A	Figure S7B	-		Figure S1
**[Tracer]**	12.5 × 10^{-9} M			Figure 4, S3C, S4	Figure S5	As in Exp. No. 1 or simulation of error *4	As in Exp. No. 1	10-point 2-fold dilution series starting from 3200 nM, 800 nM, 200 nM or 50 nM		12.5 × 10^{-9} M
**Tracer's kinetic and affinity parameters**	$k_{on} = k_1 = 2.56 \times 10^6$ $M^{-1}s^{-1}$, $k_{off} = k_2 = 1.67 \times 10^{-3}$ s^{-1}, $K_D = 6.1 \times 10^{-10}$ M			Figure 4, S3C, S4	Figure S5	. As in Exp. No. 1 or simulation of error *5	As in Exp. No. 1	Figure 5		$k_{on} = k_1 = 2.56 \times 10^6$ $M^{-1}s^{-1}$, $k_{off} = k_2 = 1.67 \times 10^{-3}$ s^{-1}, $K_D = 6.1 \times 10^{-10}$ M
**Other simulation parameters**	B_{max} = 5954.62 arbitrary binding units						[Target] = 1.0 × 10^{-10} M, reaction volume = 5 µL	Bmax = 10000 arbitrary binding units		B_{max} = 5954.62 arbitrary binding units
**Signal random scatter**	Following a double Gaussian distribution (50% with a standard deviation of 72.9 arbitrary binding units and the rest with a standard deviation of 145.8 arbitrary binding units to simulate outliers).						-	Following a double Gaussian distribution (50% with a standard deviation of 72.9 arbitrary binding units and the rest with a standard deviation of 145.8 arbitrary binding units to simulate outliers).		-

**Kinetic interval**	start: 4 s / interval: 10 s		1) start: 0 s / interval: 1 s; 2) start: 4 s / interval: 10 s, & 3) start: 0 s interval: 100 s.	start: 4 s / interval: 10 s	1) start: 4 s & interval: 10 s for 400 s or 2) start: 0 s & interval: 120 s for 4680 s	start: 4 s / interval: 10 s	interval: 10 s	interval: 10 s	interval: 10 s	Measurement start at 1 h and reading intervals of 1h
**total measurement (observation) time**	400 s	100, 200, 400, 800, 1600 and 3600 s	400 s			1600 or 400 s	400 s	400 s	400 s for association, followed by 300, 600 or 1100 s for dissociation	24 h
**Fit details**	1) subtraction of the background signal which was assumed to be linear with X (time t); 2) Fit by using method of least squares (ordinary fit) with 1000 iterations. k_1, L, k_2 and I were constrained to constant values as used in the simulation, and for the global fit k_3, k_4 and B_{max} were defined as shared parameters between the different compound concentration traces and were constrained to be greater than 0					As in Exp. No.1 BUT To assess the effect of experimental errors, the simulations (generate pseudo experimental data) were performed by using different models or input values for compound concentration, tracer concentration or tracer BK * than used for the subsequent evaluation with the Motulsky-Mahan model (analyse pseudo experimental data)		Fit by using method of least squares (ordinary fit) with 1000 iterations. Tracer concentrations were constrained to constant values as used in the simulation, and for the global fit k_{on}, k_{off} and B_{max} were defined as shared parameters between the different tracer concentration traces (where applicable: The time at which dissociation was initiated (Time0) was constrained to constant values)		1) Normalization of signals between background (0%) and tracer binding control (100%) 2) Plot log [compound] against normalised signal 3) Calculate apparent IC50 values by fitting (method: least square).
**# of Monte Carlo Experiments**	100						-	100		-

*

1) well error: simulations were performed for 2500 nM, 50 nM (or 150 nM), 25 nM and 2.5 nM compound concentrations, but compound concentrations of 2500 nM, 250 nM, 25 nM and 2.5 nM were assumed for the evaluation (only one compound concentration was assumed to be incorrect).

2) stock error: a 4-point 10-fold serial dilution of compound with a starting concentration of 3500 nM (or 2600 nM) was used for the simulation, but a 4-point 10-fold serial dilution of compound with a starting concentration of 2500 nM was assumed for the analysis with the Motulsky-Mahan model.

3) dilution error: a 12-fold (or 10.1-fold) serial dilution was emulated, but a 10-fold serial dilution was assumed for the subsequent analysis.

4) tracer concentration error: 25 nM tracer concentration were simulated, but 12.5 nM used for the Motulsky-Mahan fit (Figures S6 and S7A).

5) tracer kinetics error: a tracer on- and off-rate of either a) 2.56×10^6 M^{-1} s^{-1} and 5.02×10^3 s^{-1} or b) 2.56×10^6 M^{-1} s^{-1} and 1.67×10^2 s^{-1} or c) 7.69×10^6 M^{-1} s^{-1} and 5.02×10^3 s^{-1} or d) 7.69×10^6 M^{-1} s^{-1} and 1.67×10^3 s^{-1} was emulated, but a tracer on- and off-rate of 2.56×10^6 M^{-1} s^{-1} and 1.67×10^3 s^{-1} was assumed for the subsequent analysis step.

Supplementary Figures.

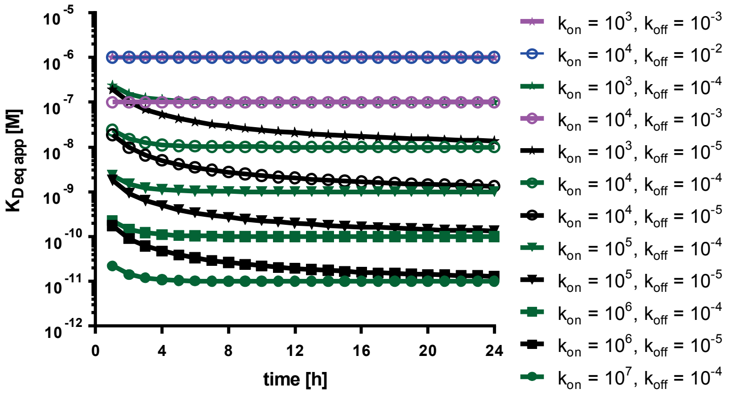

Supplementary Figure 1

Time course of apparent affinities calculated from simulated equilibrium probe competition assays (ePCA) depending on compounds binding kinetics. ePCA experiments were simulated for 12 hypothetical compounds with different on- and off-rates. All simulations considered 12.5 nM tracer with a $k_{on} = 2.56 \times 10^6$ $M^{-1}s^{-1}$ and a $k_{off} = 1.67 \times 10^{-3}$ s^{-1}. The graph shows the apparent affinities (y-axis) derived from the obtained dose-response curves after different incubation times (x-axis). Data points given with the same color or symbol come from compounds with the same dissociation rate or association rate, respectively.

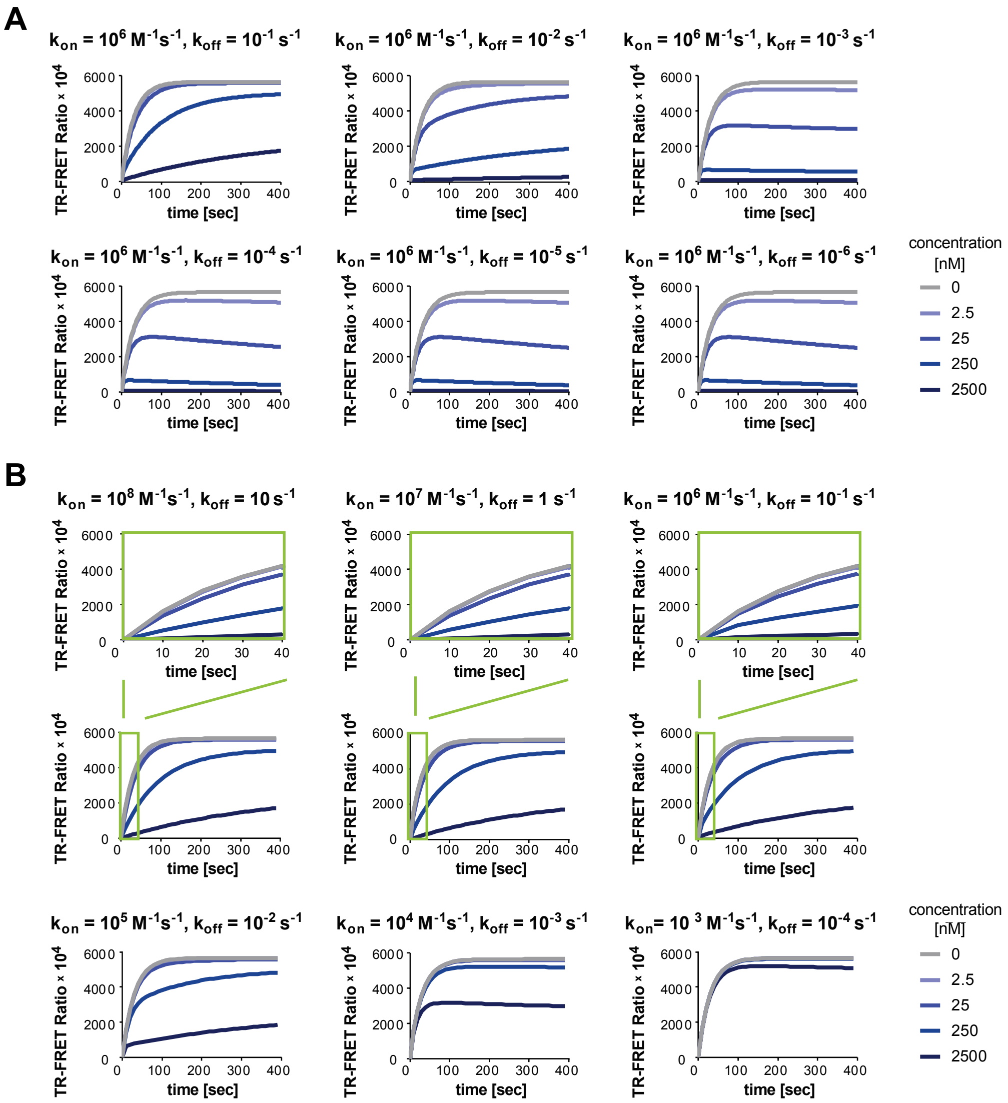

Supplementary Figure 2

Simulated time courses of competitive tracer-compound association traces illustrating the slow or the fast off-rate problem. The simulation considered an observation time of 400 s, a measuring interval of 10 s, and 12.5 nM tracer with a k_{on} = 2.56 × 10^6 $M^{-1}s^{-1}$ and a k_{off} = 1.67 × 10^{-3} s^{-1}. (A) Simulated traces for compounds with the same on-rate, but different off-rates. The lower graphs for compounds with dissociation rates of 10^{-4} s^{-1} or slower show the same binding profile. (B) Simulated traces for compounds with the same affinity, but different on- and off-rates. The upper two graphs for compounds with dissociation rates of 10^{-1} s^{-1} or faster show similar binding profiles. The top plots zoom into the first 40 seconds of the graphs below. The earliest time point (first 10 s interval) of measurement of the '250 nM compound trace' is higher for the compound with a dissociation rate of 10^{-1} s^{-1} than for those with a faster dissociation rate.

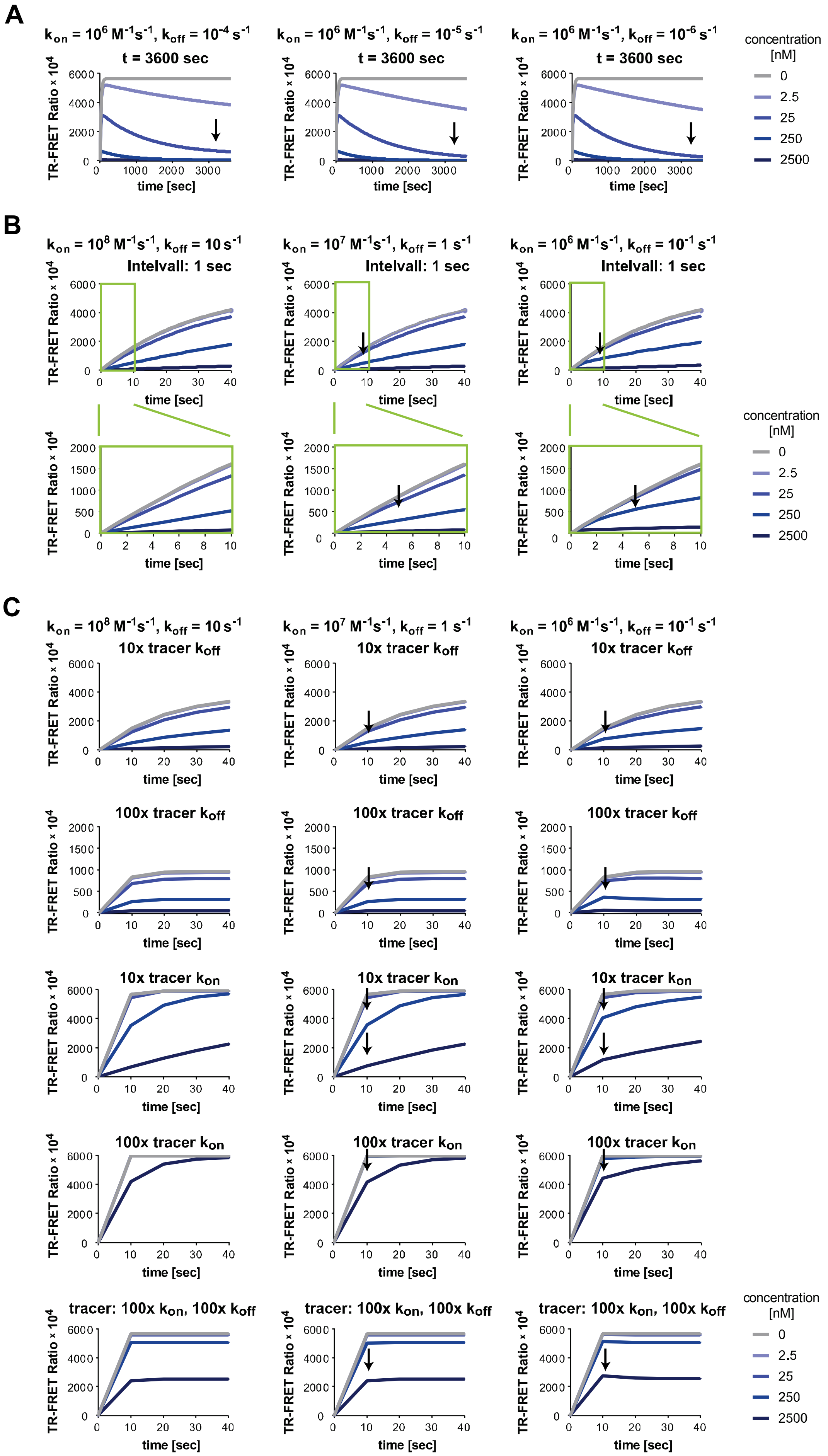

A
kon = 10^6 M^-1s^-1, koff = 10^-4 s^-1
t = 3600 sec
kon = 10^6 M^-1s^-1, koff = 10^-5 s^-1
t = 3600 sec
kon = 10^6 M^-1s^-1, koff = 10^-6 s^-1
t = 3600 sec
TR-FRET Ratio x 10^4
time [sec]
concentration [nM]
0
2.5
25
250
2500
B
kon = 10^8 M^-1s^-1, koff = 10 s^-1
Intelvall: 1 sec
kon = 10^7 M^-1s^-1, koff = 1 s^-1
Intelvall: 1 sec
kon = 10^6 M^-1s^-1, koff = 10^-1 s^-1
Intelvall: 1 sec
TR-FRET Ratio x 10^4
time [sec]
concentration [nM]
0
2.5
25
250
2500
C
kon = 10^8 M^-1s^-1, koff = 10 s^-1
kon = 10^7 M^-1s^-1, koff = 1 s^-1
kon = 10^6 M^-1s^-1, koff = 10^-1 s^-1
10x tracer koff
100x tracer koff
10x tracer kon
100x tracer kon
tracer: 100x kon, 100x koff
TR-FRET Ratio x 10^4
time [sec]
concentration [nM]
0
2.5
25
250
2500

Supplementary Figure 3

Simulated time courses of competitive tracer-compound association traces illustrating how to reduce the slow or the fast off-rate problem. If not otherwise specified, the simulations considered an observation time of 400 s, a measuring interval of 10 s, and 12.5 nM tracer with a k_{on} = 2.56 × 10^6 $M^{-1}s^{-1}$ and a k_{off} = 1.67 × 10^{-3} s^{-1}. (A) Simulated traces with an increased observation time of 1 h for compounds with the same on-rate, but different off-rates. The arrows indicate the decreasing area under the curve of the '250 nM compound trace' with slower dissociation rates, which can be observed at the end of the observation period. (B) Simulated traces with a faster measuring interval of 1 s for compounds with the same affinity, but different on- and off-rates. The bottom plots zoom into the first 10 seconds of the graphs above. At the early time points (1-10 s) of measurement the area under curves are larger for the compound with a dissociation rate of 10^{-1} s^{-1} than for those with a faster dissociation rate. (C) Simulated traces with different tracer binding kinetics (as indicated above the graphs) for compounds with the same affinity, but different on- and off-rates. The arrows indicate the higher signals at the earliest time point (first 10 s interval) of measurement with decreasing dissociation rate of the compound.

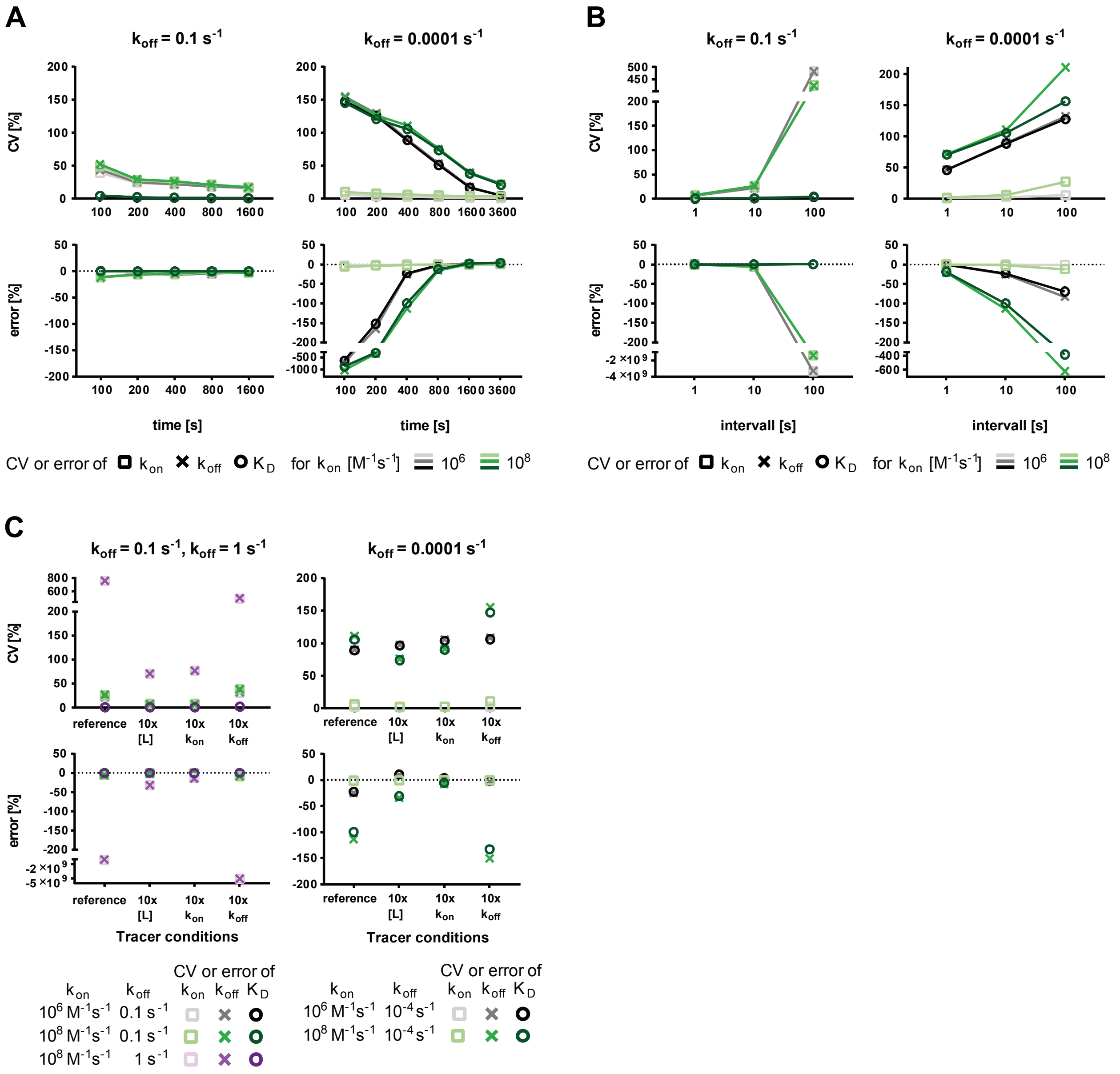

Supplementary Figure 4

Precision (CV) and accuracy (relative error) of the Motulsky-Mahan model depending on observation time (A), measuring interval (B) and tracer characteristics (C). If not otherwise specified, the simulations considered an observation time of 400 s, a measuring interval of 10 s, and 12.5 nM tracer with a k_{on} = 2.56×10^{6} $M^{-1}s^{-1}$ and a $k_{off} = 1.67 \times 10^{-3}$ s^{-1}. Panel A, B and C show the results from the analysis in Figures 2B, 3B and 4B, respectively, where the simulations were performed for a fast (10^{-1} s^{-1}) and a slower dissociating (10^{-4} s^{-1}) compound, both with an association rate of 10^{6} $M^{-1}s^{-1}$ (grey). Additionally, panel A, B and C present the result for the same analysis, but with an alternative association rate of 10^{8} $M^{-1}s^{-1}$ (green). Moreover, panel C depicts CVs and the relative errors determined from a similar Monte Carlo analysis, but for a faster dissociating (1 s^{-1}) compound with an association rate of 10^{8} $M^{-1}s^{-1}$ (purple).

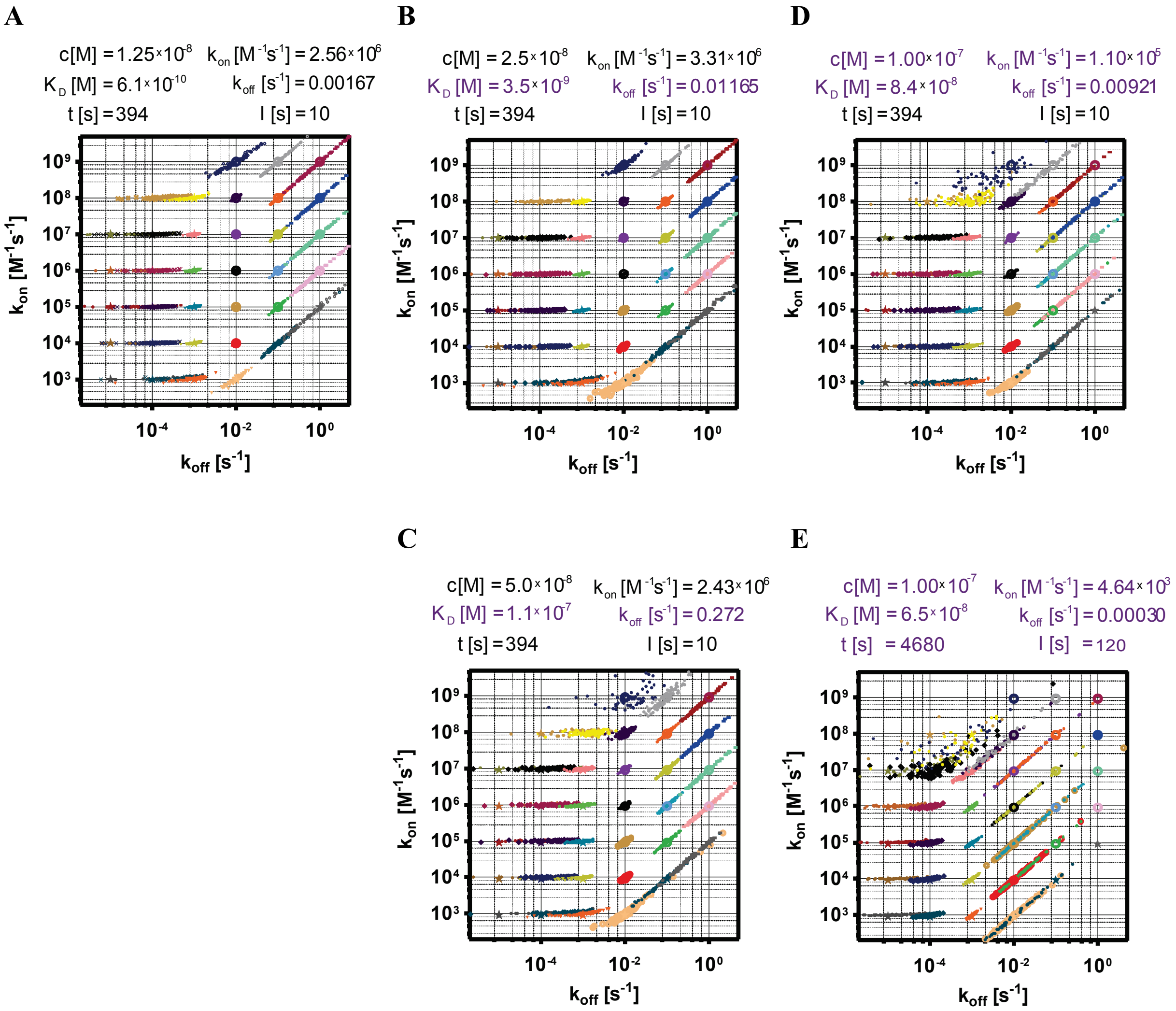

Supplementary Figure 5

Performance of the Motulsky-Mahan model depending on tracer and compound binding kinetics, tracer concentration, incubation time and measuring interval. Similar to the analysis in Figure 1, Monte Carlo simulations and analyses (100 simulations of kinetic probe competition experiments, respectively) were performed for 35 hypothetical compounds with different on- and off-rates. The simulations considered an observation time t, a measuring interval I, a tracer concentration c, and tracer binding kinetics (k_{on}, k_{off}) as indicated above the rate plots in panel A-E. The rate plots represent the input binding kinetic parameters (large symbols) for the 35 compounds (one color per compound) as used for the simulation along with the corresponding output rates (small symbols) calculated by using the Motulsky-Mahan model. The diagonals in the plots are isoaffinity lines. All plots zoom into the range of the input parameters. Thus, not all output parameters are inside the axis limits.

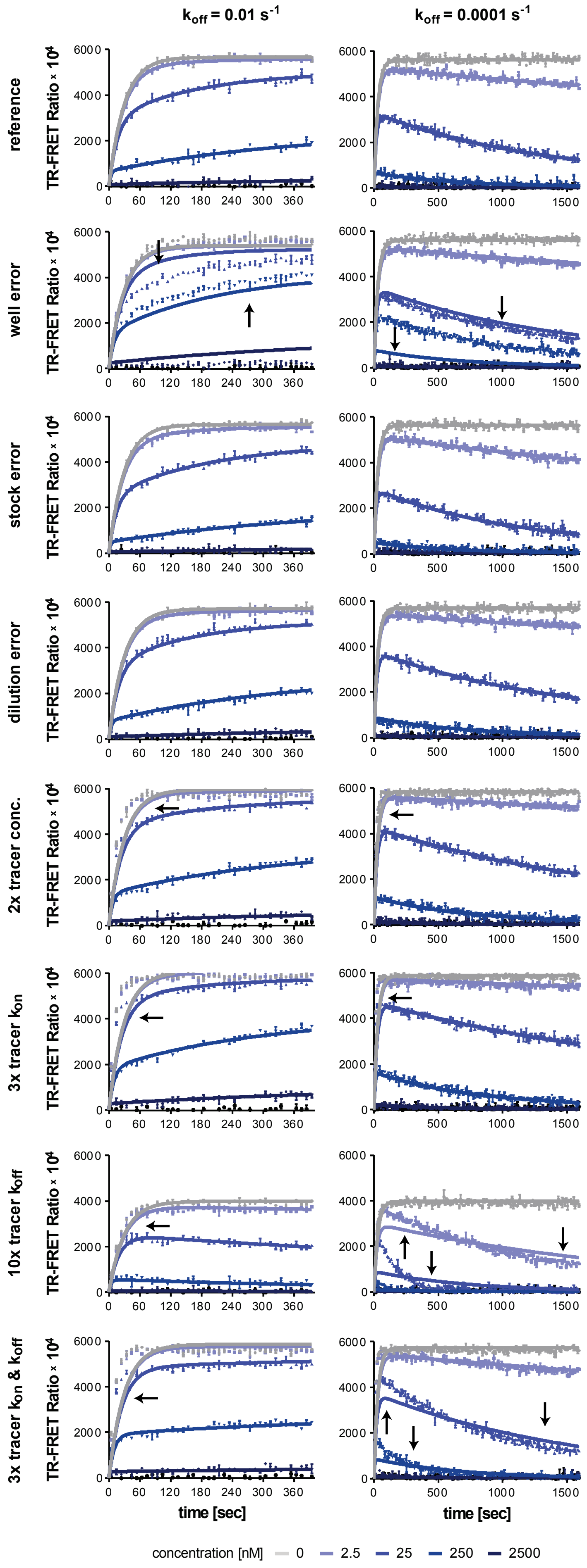

Supplementary Figure 6

Effect of experimental errors on simulated time courses of competitive tracer-compound association traces and the corresponding global fit of the Motusky-Mahan equation to the data points.

The reference simulations (top panels) considered a measuring interval of 10 s, a 4-point 10-fold serial dilution of compound with a starting concentration of 2500 nM and 12.5 nM tracer with a k_{on} = 2.56 × 10^6 $M^{-1}s^{-1}$ and a k_{off} = 1.67 × 10^{-3} s^{-1}. The observation time was 400 s for the faster dissociating compound (k_{on} = 10^6 $M^{-1}s^{-1}$, k_{off} = 10^{-2} s^{-1}) (left), and 1600 s for the slower dissociating compound (k_{on} = 10^6 $M^{-1}s^{-1}$ and k_{off} = 10^{-4} s^{-1}) (right).

The rows below present the signal traces simulated experimental errors and the corresponding fits. The arrows indicate the deviations between the best-fit curves and the corresponding signal trace. The well error is a wrong compound concentration in a single well (50 nM instead of 250 nM). The stock error simulation assumes a wrong compound stock concentration used for the serial dilution of compound (3500 nM instead of 2500 nM as considered in the Motulsky-Mahan model fit). The dilution error refers to a 12fold instead of a 10fold serial dilution of the compound. The other rows show the effect of errors in tracer concentration or resulting from wrongly determined tracer binding kinetics.

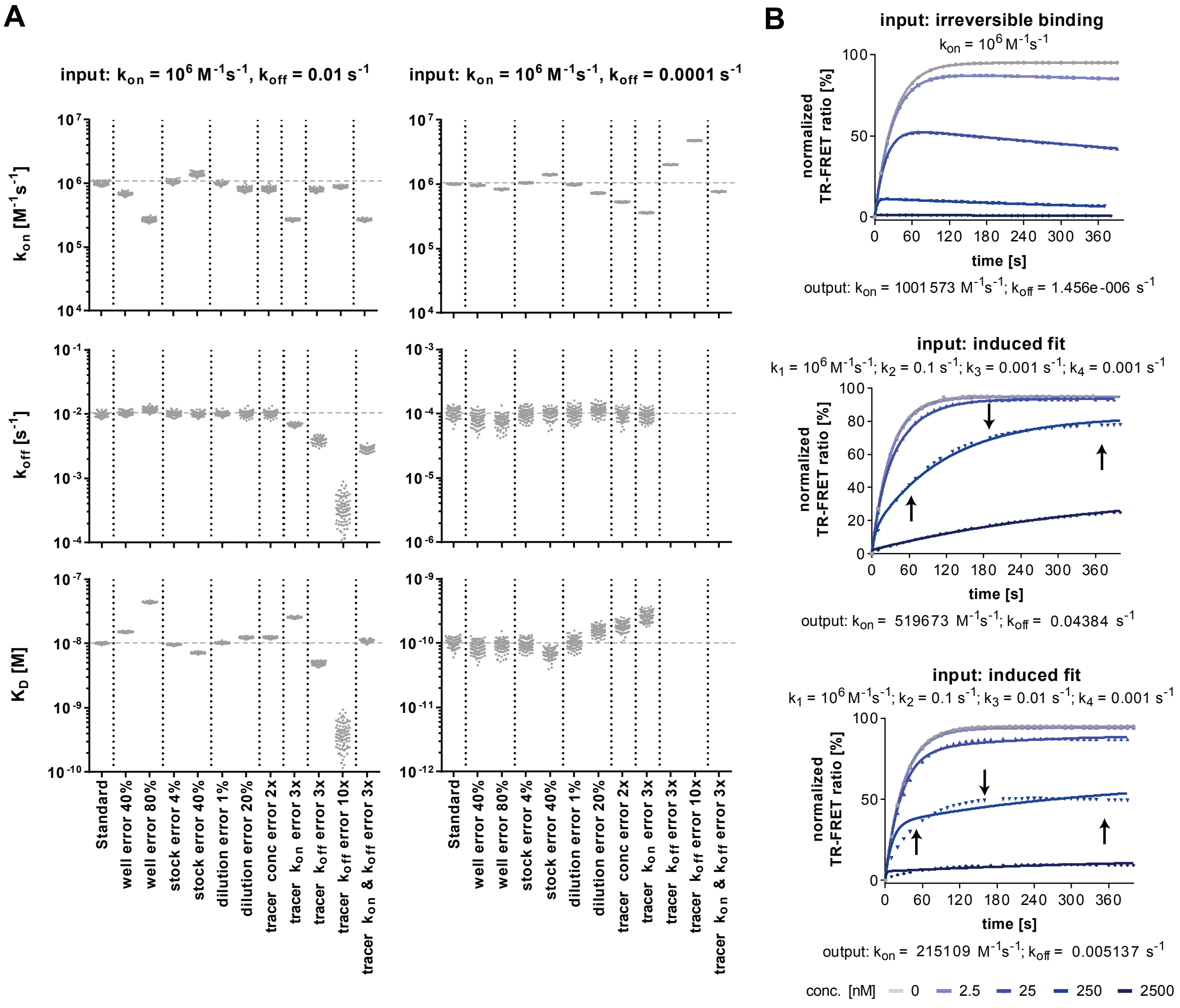

Supplementary Figure 7

Effect of experimental and conceptual errors on the results of kinetics of competitive binding experiments. (A) The effect of experimental errors on accuracy. Monte Carlo simulations and analyses similarly or identical to those shown in Supplementary Figure 6 were performed 100 times, respectively. The graphs represent the input binding kinetic parameters (horizontal dashed line) along with the output parameters (grey dots) calculated by using the Motulsky-Mahan model. Not all output parameters are inside the y-axis limits. (B) The effect of conceptual errors on simulated time courses of competitive tracer-compound association traces and the corresponding global fit of the Motusky-Mahan equation to the data points. The simulations considered an observation time of 400 s, a measuring interval of 10 s, 12.5 nM tracer with a k_{on} = 2.56×10^6 $M^{-1}s^{-1}$ and a k_{off} = 1.67×10^{-3} s^{-1}, and a compound binding irreversible or via induced fit to the target molecule. However, the model equation used for the analysis assumes a simple 1:1 binding model. The arrows indicate the deviations between the best-fit curves and the corresponding signal trace.
